# Supplementary material for: Healthy Dads, Healthy Kids UK, a weight management programme for fathers: feasibility RCT
Source: BMJ Open. 2019 Dec 10;9(12):e033534. doi: 10.1136/bmjopen-2019-033534 (PMC6924741; doi:10.1136/bmjopen-2019-033534)
Supplement: Supplementary data [file bmjopen-2019-033534supp001.pdf]

Web-table 1: The HDHK UK programme reported according to the Template for Intervention Description and Replication (TIDieR) checklist

Item	Item description
1. Name	Healthy Dads, Health Kids UK
2. Why	Following the success of the Healthy Dads, Healthy Kids programme in Australia the aim of adapting the programme was to test the suitability of the programme to an ethnically diverse UK population.<br><br>The primary aim of adapting the HDHK programme was to increase acceptability of the programme to a socio-economically disadvantaged, ethnically diverse UK population.
3 & 4: Materials and Procedures	Fathers of primary school aged children who were classed as overweight or obese (BMI >25kg/m^2 (>23kg/m^2 for fathers of South Asian origin) with primary school aged children (4-11 years) were invited to take part in the HDHK study. Participants randomised to the intervention group were asked to attend the HDHK intervention programme with their children. The intervention programme involved a 90 minute session once a week for nine weeks with weekly goals and activities to be completed in their own time<br><br>**<u>Materials provided to the participants</u>**<br><br>Fathers and children were provided with an intervention pack:<br><br>**Fathers:**<br>- A handbook to accompany the father-only education sessions<br>- A logbook for monitoring of weekly activities including step counter, weight chart etc.<br>- A HDHK branded black T-shirt<br>- Nine 'Green slips' - cards with weekly activities to be completed either on their own, with their children or as a family<br>- A certificate (presented by their children) on completion of the programme<br><br>**Children:**<br>- A workbook to work through as part of the children-only education sessions. This included weekly activities to complete with their father<br>- Activity cards with instructions how to practice fundamental movement skills and a spinner (as part of a game to select which card to pick each day)<br>- A HDHK branded green T-shirt<br>- A certificate (presented by facilitators) on completion of the programme<br><br>**<u>Materials provided to the facilitators</u>**<br>- HDHK branded red T-shirt<br>- Facilitators guide to intervention delivery

Griffin T, *et al. BMJ Open* 2019; 9:e033534. doi: 10.1136/bmjopen-2019-033534

	- Father-only education session facilitators were provided with the PowerPoint slides for delivery<br>- Access to on-line demonstrations of the physical activity components of the programme<br>- Exercise<br><br>Each week the 90 minute course followed the same format:<br><br>**0-15 minutes. Father and children review of their weekly goals with facilitators**<br><br>- A review session where participants and their children updated the facilitators on their activity and if they had completed their weekly tasks the children were given a sticker for their workbooks<br>- This section also included a father-child bonding activity where they were encouraged to talk to each other or play a short game such as ‘thumb war’ or guess each other’s favourite movie/food/activity.<br>- Fathers had opportunity to use scales to weigh themselves and complete their log book<br><br>**15-45 minutes. Fathers (‘father-only’) and children (‘children-only’) participate in separate education sessions**<br><br>- Father only sessions were delivered by the facilitator using a pre-prepared PowerPoint slide show which was delivered encouraging discussion around the key points. There were also additional activities each week to be completed in the logbook such as setting goals (weight, physical activity of nutrition goals), calculating daily energy needs,<br>- Every week fathers were asked to report on their weight and average number of daily steps (aiming to 10,000 steps/day)<br>- Child-only education sessions were delivered by working through the children’s workbook. The session content followed that of the father sessions but made simple to be child appropriate.<br>- Children were also asked to select two activities each week to complete with their fathers. The activities were planned around physical activity or nutrition, such as ‘how many press ups can Dad do in 1minute’ , ‘set up an obstacle course’, ‘get Dad to drink at least 8 glasses of water a day’.<br><br><u>Fathers’ sessions:</u><br><br>Week 1: Dads matter in children’s health - Highlights the unique influence of dads in contributing to the physical and mental health of children.<br><br>Week 2: Weight management for men - Explores the challenges of healthy eating in the modern world, outlines the mathematics of weight loss and setting SMART goals to achieve activity and dietary ambitions.<br><br>Week 3: Being a healthy dad – Strategies to enhance your and your family’s life. Highlights 9 weight loss tips for men, tells dads

Griffin T, *et al. BMJ Open* 2019; 9:e033534. doi: 10.1136/bmjopen-2019-033534

	how to 'stay on track' and provides advice on sustainable approaches to weight loss.<br><br>Week 4: Healthy eating for families (Mums attend) - Provides advice on appropriate portion sizes for the whole family, discusses strategies for implementing the trust paradigm to encourage their children to eat healthily at home.<br><br>Week 5: The unique and powerful influence of fathers - Explains to dads why they have such a powerful influence over their kids, the importance of being a good role model and outlines the most effective parenting style.<br><br>Week 6: Raising active kids in an inactive world - Explains the growing issues of childhood obesity and why physical activity is so important for kids, highlights key strategies for dads to be physical activity leaders.<br><br>Week 7: 'Switching on' your child's mind by 'Switching off' - Highlights the physical and mental health issues created by excessive screen time and provides strategies for 'switching off'.<br><br>Week 8: 'Healthy' fathering in a busy world - Encourages discussion of barriers and solutions for achieving SMART goals, highlights opportunities to create family traditions and maximize the time dads can spend with their kids.<br><br>Children's sessions:<br><br>Week 1: Rough and tumble play - children learn about their mission to 'get dad fit and healthy' and are taught about rough and tumble activities.<br><br>Week 2: Turning Dad into a healthy eater - Through fun activities, kids learn about 'sometimes' foods and 'anytime' foods and how they can encourage dad to eat more healthily.<br><br>Week 3: Helping dad make healthy choices - children came up with suggestions for their Dad's packed lunch and were introduced to goal setting.<br><br>Week 4: The HDHK rainbow plate - Through fun activities, children learn about different fruits and vegetables and are challenged to make their plates 'rainbows' with a variety of healthy fruits and vegetables.<br><br>Week 5: Fun times with Dad - children are given activities to help them think about games they can play with dad to spend quality time together.<br><br>Week 6: Let's get active - children learning about importance of being active and considering how to stay active.<br><br>Week 7: Helping Dad 'Switch off' - children think about activities they could enjoy with Dad instead of playing on the computer or watching TV.

Griffin T, *et al. BMJ Open* 2019; 9:e033534. doi: 10.1136/bmjopen-2019-033534

	Week 8: Becoming Dad's personal trainer - children develop an activity board with games and exercises the family can complete at home.<br>Week 9: Helping dad stay on track<br>**45-90 minutes. Fathers and children joint physical activity session**<br>Each week the joint father and child physical activity session covered three components:<br>- Rough and Tumble Play – *hands on activities played by each participant with their child, such as 'sock wrestle' (trying to get each other's socks off), 'stand up Dad' (children would try to stop their Dad standing up)*<br>- Fundamental skill development (for children) – *Coached activities focusing on child skill development of FMS such as catching, throwing, jumping, running etc.*<br>- Fitness activity (for fathers) – *Fitness activity focusing on fathers' fitness with support from children (e.g. Child helps count Dad's press ups, or sits on Dads back to make it harder or family running games racing each other).*
5. Who provided?	Three facilitators were required to deliver the HDHK programme. One facilitator to deliver the father-only education sessions and two facilitators to deliver to child-only education and joint father and child activity sessions.<br>Staff were recruited from four organisations to deliver the programme across the different courses. A face to face training session, a course manual and all materials required for delivery were provided.<br>The fathers-only education session facilitators were delivered by:<br>- Local Authority Healthy lifestyle Team (health trainer - female) – course 1<br>- Leisure centre staff employees (health and lifestyles manager and gym manager – both male) – course 2&3<br>- Sports coach of a national coaching organisation (female) – course 4<br>For successful delivery father-education facilitators were required to have presentation and group facilitation skills. The programme was designed for this section to be delivered by male facilitators, however due to staff availability this was not always possible.<br>The child-only education sessions and joint child and family activity sessions were delivered by:<br>- Self-employed fitness instructor - course 1<br>- Sports coaches – courses 2, 3 & 4.<br>Whilst not essential for delivery, sports coaching skills supported successful delivery to the physical activity elements of the programme. For child protection and ease of facilitation it was recommended that two facilitators ran the child-only education sessions. The second facilitator does not need to lead the session

	and this role could be undertaken by research staff or work experience students.
6: How	HDHK-UK is a group based programme, optimum group size 8-15 fathers
7. Where?	The programme is delivered in community venues such as community, youth or leisure centres and schools. The venue must have classroom (or equivalent) space for an educational session with PowerPoint projection facilities and a sports hall (or equivalent) for the physical activity session.
8. When and how often?	The programme is 9-weeks long with one 90-minute session delivered each week. Sessions need to be delivered at a time fathers and their children (aged 4-11 years) can attend. Of the four courses delivered, two were on a weekday evening, two were on a weekend. The weekend sessions were more popular.
9. Tailoring	Each week throughout the education session fathers were encouraged to reflect on the session content and apply the key messages in context to their own personal circumstances. Similarly, participants were asked to tailor their weekly goals and set home activities to meet their own and their family needs.
Modifications	Details of the programme adaptation from the original Australian programme are presented earlier in this chapter. Throughout the delivery of the adapted programme small modifications were made between courses, mainly where delivery of the father-only education session took longer than the allocated 30 minutes the material was shortened to ensure there was sufficient time for the physical activity session.
How well the programme was delivered -planned	The university research team completed observations of intervention delivery, completing a checklist to report on items of fidelity, including session timings, the delivery of key messages, facilitator skills and enthusiasm and participants responsiveness.
How well the programme was delivered - actual	Researcher observations of programme delivery showed that it was delivered with good fidelity. The main challenge was keeping the father-only education session to the allotted 30-minutes. This was improved throughout the courses. The fidelity of intervention delivery is described in Chapter 6.

Griffin T, *et al. BMJ Open* 2019; 9:e033534. doi: 10.1136/bmjopen-2019-033534
