# Supplementary material for: Healthy Dads, Healthy Kids UK, a weight management programme for fathers: feasibility RCT
Source: BMJ Open. 2019 Dec 10;9(12):e033534. doi: 10.1136/bmjopen-2019-033534 (PMC6924741; doi:10.1136/bmjopen-2019-033534)
Supplement: Supplementary data [file bmjopen-2019-033534supp002.pdf]

Web-table 2: Baseline characteristics for the completers and non-completers at 6 months

	**Non-completers at 6 months** (N=16)	**Completers at 6 months** (N=27)	**Overall** (N=43)
**Minimisation Variables**			
Ethnicity			
White British	10 (62.5%)	7 (25.9%)	17 (39.5%)
Non White British	6 (37.5%)	20 (74.1%)	26 (60.5%)
**Demographics**			
Age (years)			
Mean (SD), N	38.7 (7.7), 16	40.7 (5.5), 27	40.0 (6.4), 43
Minimum-Maximum	23.6-56.0	31.6-52.8	23.6-56.0
Weight (kg)			
Mean (SD), N	93.4 (16.0), 16	89.2 (14.9), 27	90.8 (15.3), 43
Minimum-Maximum	70.9-136.9	64.0-142.5	64.0-142.5
BMI (kg/m^2)			
Mean (SD), N	30.9 (6.1), 16	29.7 (4.4), 27	30.2 (5.1), 43
Minimum-Maximum	25.1-50.9	23.8-45.5	23.8-50.9
Index of Multiple Deprivation quintile, n (%)			
1 (least deprived)	1 (6.3%)	0 (0.0%)	1 (2.3%)

Griffin T, *et al. BMJ Open* 2019; 9:e033534. doi: 10.1136/bmjopen-2019-033534

	**Non-completers at 6 months** (N=16)	**Completers at 6 months** (N=27)	**Overall** (N=43)
2	2 (12.5%)	0 (0.0%)	2 (4.7%)
3	2 (12.5%)	4 (14.8%)	6 (14.0%)
4	2 (12.5%)	7 (25.9%)	9 (20.9%)
5 (most deprived)	9 (56.3%)	14 (51.9%)	23 (53.5%)
Missing	0 (0.0%)	2 (7.4%)	2 (4.7%)

Note: All figures presented are N (%) unless otherwise specified.

Griffin T, *et al. BMJ Open* 2019; 9:e033534. doi: 10.1136/bmjopen-2019-033534
