# Supplementary material for: Healthy Dads, Healthy Kids UK, a weight management programme for fathers: feasibility RCT
Source: BMJ Open. 2019 Dec 10;9(12):e033534. doi: 10.1136/bmjopen-2019-033534 (PMC6924741; doi:10.1136/bmjopen-2019-033534)
Supplement: Author's manuscript [file bmjopen-2019-033534.draft_revisions.pdf]

BMJ Open is committed to open peer review. As part of this commitment we make the peer review history of every article we publish publicly available.

When an article is published we post the peer reviewers' comments and the authors' responses online. We also post the versions of the paper that were used during peer review. These are the versions that the peer review comments apply to.

The versions of the paper that follow are the versions that were submitted during the peer review process. They are not the versions of record or the final published versions. They should not be cited or distributed as the published version of this manuscript.

BMJ Open is an open access journal and the full, final, typeset and author-corrected version of record of the manuscript is available on our site with no access controls, subscription charges or pay-per-view fees (http://bmjopen.bmj.com).

If you have any questions on BMJ Open's open peer review process please email info.bmjopen@bmj.com

BMJ Open

A weight management programme for fathers: Healthy Dads, Healthy Kids UK – a feasibility RCT

Journal:	*BMJ Open*
Manuscript ID	bmjopen-2019-033534
Article Type:	Original research
Date Submitted by the Author:	09-Aug-2019
Complete List of Authors:	Griffin, Tania; University of Bath, Department for Health<br>Sun, Yongzhong; University of Birmingham, Birmingham Clinical Trials Unit (BCTU)<br>Sidhu, Manbinder S.; University of Birmingham, Health Services Management Centre<br>Adab, Peymane; University of Birmingham, Institute of Applied Health Research<br>Burgess, Adrienne; Fatherhood Institute<br>Collins, Clare; University of Newcastle, NSW, AU, School of Health Sciences<br>Daley, Amanda; Loughborough University, School of Sport, Exercise and Health Sciences<br>Entwistle, Andrew; Patient/Carer Reviewer,<br>Frew, Emma; University of Birmingham, Institute of Applied Health Research<br>Hardy, Pollyanna ; University of Birmingham, Birmingham Clinical Trials Unit<br>Hurley, Kiya; University of Birmingham, Cancer Research UK Clinical Trials Unit<br>Jones, Laura; University of Birmingham, Institute of Applied Health Research<br>McGee, Eleanor ; Birmingham Community Healthcare NHS Trust<br>Pallan, Miranda; University of Birmingham, Institute of Applied Health Research<br>Young, Myles; University of Newcastle, NSW, AU, School of Education<br>Morgan, Philip; University of Newcastle, NSW, AU, School of Education<br>Jolly, Kate; University of Birmingham, Institute of Applied Health Research
Keywords:	Feasibility studies, Fathers, Weight loss, Healthy lifestyle

SCHOLARONE™
Manuscripts

A weight management programme for fathers: Healthy Dads, Healthy Kids UK – a feasibility RCT

Authors:

Tania Griffin[1], Yongzhong Sun[2], Manbinder Sidhu[3], Peymane Adab[4], Adrienne Burgess[5], Clare Collins[6], Amanda Daley[7], Andrew Entwistle[8], Emma Frew[4], Pollyanna Hardy[2], Kiya Hurley[9], Laura Jones[4], Eleanor McGee[10], Miranda Pallan[4], Myles Young[11], Philip J Morgan[11], Kate Jolly[4],

Corresponding author: Kate Jolly

1. Department for Health, University of Bath, Bath, UK
2. Birmingham Clinical Trials Unit, Institute of Applied Health Research, University of Birmingham, Birmingham, UK
3. Health Services Management Centre, University of Birmingham, Birmingham, UK
4. Institute of Applied Health Research, University of Birmingham, Birmingham, UK
5. The Fatherhood Institute, Marlborough, Wiltshire, UK
6. School of Health Sciences, Faculty of Health and Medicine, and Priority Research Centre for Physical Activity and Nutrition, University of Newcastle, New South Wales, Australia
7. School of Sport, Exercise and Health Sciences, Loughborough University, Loughborough, UK
8. Sandiacre, Sandy Lane, Leamington Spa, CV32 6QS
9. Cancer Research UK Clinical Trials Unit, Institute of Cancer and Genomic Sciences, University of Birmingham, Birmingham, UK
10. Birmingham B15 2TT Birmingham Community Health Care NHS Foundation Trust, Birmingham B7 4BN, UK
11. School of Education, and Priority Research Centre for Physical Activity and Nutrition, University of Newcastle, New South Wales, Australia

Keywords: Feasibility Studies, Fathers, Weight loss, Healthy Lifestyle

Word Count: 4000

For peer review only - http://bmjopen.bmj.com/site/about/guidelines.xhtml

ABSTRACT

Objectives: To assess the feasibility of conducting a randomised controlled trial (RCT) evaluating the Healthy Dads, Healthy Kids United Kingdom (HDHK-UK) programme for fathers with overweight or obesity and their primary school aged children.

Design: A two-arm, randomised feasibility trial with a mixed-methods process evaluation.

Setting: Socio-economically disadvantaged, ethnically diverse localities in West Midlands, UK.

Participants: Fathers with overweight or obesity and their 4-11 year old children.

Intervention: Participants were randomised in a 1:2 ratio to control (family voucher for a local leisure-centre) or intervention comprising nine weekly healthy lifestyle group sessions (education and practical physical activity).

Outcomes: Feasibility of the intervention and RCT were assessed according to pre-specified progression criteria: study recruitment, consent and follow-up, ability to deliver intervention, intervention fidelity, adherence and acceptability, weight loss, using questionnaires and measurements at baseline, 3 and 6-months, and through qualitative interviews.

Results: The study recruited 43 men, 48% of the target sample size; mean BMI 30.2kg/m^2 (SD 5.1), 61% were from a minority ethnic-group and 54% from communities in the most disadvantaged quintile for socio-economic deprivation. Recruitment was challenging. Retention at 3 and 6-month follow-up was 63%. Identifying delivery sites and appropriately skilled and trained programme facilitators proved difficult. Four programmes were delivered in leisure-centres and community venues. Of the 29 intervention participants, 20 (69%) attended the intervention at least once, of whom 75% attended ≥5 sessions. Sessions were delivered with high fidelity. Participants rated sessions as 'good/very good' and reported lifestyle behavioural change. Weight loss at 6 months in the intervention group (n=17) was 2.9kg (95%CI -5.1 to -0.6).

Conclusions: The intervention was well received, but there were significant challenges in recruitment, programme delivery and follow-up. The HDHK-UK study was not considered feasible for progression to a full RCT based on pre-specified stop-go criteria.

Trial registration: ISRCTN16724454

For peer review only - http://bmjopen.bmj.com/site/about/guidelines.xhtml

Strengths and limitations of this study

- This is a study of weight management programme for fathers from a socio-economically deprived, ethnically diverse community setting, a unique study given the relative paucity of research in this area.
- Recruitment methods were wide ranging, raising awareness of the study in a number of community settings, however despite extensive efforts recruitment to the study was a key challenge.
- The intention was to recruit male facilitators to deliver the intervention sessions, however it was difficult to identify suitable facilitators with the necessary skill set, therefore a range of male and female facilitators were recruited.
- Identifying a convenient time to run the intervention course to suit fathers, venue and facilitator availability proved challenging.

For peer review only

For peer review only - http://bmjopen.bmj.com/site/about/guidelines.xhtml

INTRODUCTION

Overweight and obesity remain a public health challenge and priority.[1] At an individual level excess weight results in fewer disease-free years and lowered life expectancy,[2, 3] and at a population level obesity prevalence is associated with major economic burden.[4]

In England, overweight and obesity levels are higher in men compared to women with socioeconomic inequalities evident.[5] Men from the lowest income quintile have a greater mean waist circumference than men from the highest income quintile.[6] South Asian men have higher body fat percentage than White Europeans, at the same body mass index (BMI).[7]

The transition to fatherhood has been identified as a critical period during which men are more likely to adopt obesogenic behaviours and are more susceptible to weight gain.[8, 9] While there have been several successful men-only weight management programmes,[10-14] attendance for such activities remains higher amongst women.[13] Fathers are also under-represented in interventions targeting family health behaviours.[15] This emphasises the need to identify programmes which fathers are keen to engage with.

Healthy Dads Healthy Kids (HDHK) is a weight management programme for fathers of primary school aged children that also addresses the healthy eating and physical activity behaviours of their children. The programme, developed and delivered in Australia, was shown to be successful at achieving weight loss in fathers.[16, 17] In the current study, the HDHK programme was adapted with the intention of making it culturally acceptable to a multi-ethnic UK population.[18] The aim of this study was to assess the feasibility of delivering the adapted HDHK programme and the feasibility to conduct a future RCT in a socioeconomically deprived ethnically diverse UK setting.

METHODS

Study design and participants

This study was a two-arm, randomised feasibility trial with a mixed-methods process evaluation. It was conducted in two urban local authority areas of the West Midlands, UK, (site 1 and site 2). In 2017, both areas were ranked in the most deprived 20% of areas in the UK, with high ethnic diversity.[19]

The study aimed to recruit men who were father/step-father/father figures (herein referred to as ‘fathers’) of primary school children (aged 4-11 years), aged 18-65 years with a BMI ≥25kg/m^2 (≥23kg/m^2 for minority ethnic groups)[7] and/or a waist circumference of ≥94cm (37

For peer review only - http://bmjopen.bmj.com/site/about/guidelines.xhtml

inches) who were willing to lose weight. Fathers did not have to be co-resident with their child(ren). Men were not eligible if they had a history of angina or other cardiovascular disease; orthopaedic or joint problems that would be a barrier to vigorous physical activity; weight loss of 3kg/7lbs in the previous three months; current diabetes and were not confident in managing their condition during exercise; were unable to speak and/or understand English; involved in ongoing custody or access disputes and/or any contexts with a risk of domestic violence.

Sample size

As a feasibility study, no formal sample size calculation was performed. We aimed to recruit 90 men and their children to estimate the recruitment, follow-up and questionnaire completion rates to within +/- 10% with 95% confidence, based on a worst case estimate of 50%.

Recruitment and randomisation

Fathers were recruited (September 2017-January 2018) using a range of methods including flyer distribution and promotion stands at leisure, community and shopping centres, places of worship and large workplace organisations. Recruitment via schools conducted through presentations at school assemblies and teacher meetings, stands at parent evenings, flyer distribution and talking to parents at school pick up time. The study was promoted on social media (Twitter and Facebook).

Written informed consent was obtained from fathers and assent from children aged 8 years and over. Once baseline data were collected, participants were randomised (1:2 allocation ratio) to the control group (voucher for a single family visit to a leisure centre) or intervention group (adapted HDHK-UK programme). Randomisation was stratified by father's ethnicity (white British or Irish / other ethnic group) and conducted using an automated online form developed by University of Birmingham Clinical Trials Unit.

Intervention

The HDHK-UK intervention comprised weekly 90-minute sessions over nine consecutive weeks; four courses were delivered. Fathers and children attended all sessions which followed the same structure: 15 minutes' discussion and review of the weekly activities followed by 30 minutes where children and fathers took part in an education session separately. Fathers' sessions covered a range of lifestyle behaviours around the importance of physical activity, nutrition and parenting. The group was facilitated by local, experienced and trained staff to ensure the sessions were interactive and discussion was encouraged. Children were taught about healthy eating, physical activity and how to be a supportive family member by encouraging and modelling healthy lifestyle behaviours at home. The final 45 minutes of the

For peer review only - http://bmjopen.bmj.com/site/about/guidelines.xhtml

session were spent doing physical activity within family groups. These practical sessions had three elements: 'rough and tumble' play; teaching children fundamental movement skills (catching, throwing, kicking etc.); and aerobic fitness. The intervention is summarised in TIdIER checklist (web-table 1). The HDHK intervention draws on concepts from Family Systems Theory[20, 21] and Social Cognitive Theory; [22] the theoretical constructs are reported for the original programme.[17] Adaptations to the Australian resources were made by the research team (KJ) and one of the study partners - The Fatherhood Institute. The adaptations focussed on reducing the number of PowerPoint slides, simplifying and anglicising wording and updating the guidance and statistics to align to UK public health recommendations. References to foods, activities and images were updated to reflect a multi-cultural UK population. Fathers and their children attended every session and mothers were invited on one occasion when family food was discussed.

Data collection and outcomes

Data were collected from participants by trained researchers at baseline, and three- and six-months later in their home (or convenient location). Sociodemographic characteristics were collected by questionnaire. Based on postal code of residence, the Index of Multiple Deprivation (IMD) was used to determine socioeconomic status (SES).[23]

Primary outcomes: feasibility measures

The outcomes relating to feasibility of delivery of HDHK-UK were: ability to recruit and retain facilitators, deliver sessions at a time and location convenient for participants, fidelity of delivery, and acceptability to participants.

The outcomes relating to the feasibility of conducting a future definitive RCT were: recruitment rate, willingness to be randomised, follow-up rates, level of completion of follow-up questionnaires, and father's weight change in the intervention group.

Process evaluation

Process measures were collected by the research team to determine the feasibility of intervention delivery and study processes, fidelity of intervention delivery and participant acceptability. Sixteen session observations (minimum of two per intervention course) were completed to assess content delivery and participant engagement. Participants and facilitators were also asked to complete feedback forms after each session, evaluating the session content and delivery. Qualitative interviews were conducted with 12 participants post-intervention. One participant was interviewed at both 3 and 6-months. The average interview duration was 16 minutes and interviews were conducted either face to face (n=4) or by

For peer review only - http://bmjopen.bmj.com/site/about/guidelines.xhtml

telephone (n=9). All facilitators (n=7) were interviewed, five interviews were conducted individually, one with two facilitators together.

Secondary outcome measures

Secondary outcome measures for fathers were outcomes that would be collected in the main trial (if progressed to). These comprised: percentage losing ≥5% body mass, change in waist circumference and percent body fat, self-reported physical activity (using the IPAQ-short[24]), objectively measured physical activity (by a wrist worn GeneActiv© (Activinsights, Cambs, United Kingdom), triaxial accelerometer worn for 7-days on the father's non-dominant wrist), self-reported dietary intake (using food frequency items (FFQ) items), father-child relationship outcomes (using the Parent-Child Relationships Questionnaire),[25] parenting for physical activity (using the Parenting Strategies for Eating and Activity Scale[26] and the Physical Activity Modelling subscale of the Activity Support Scale for Multiple Groups (ACTS-MG)[27]).

Secondary outcome measures collected from the children comprised BMI z-score change (calculated using the LMS method[28] and UK reference data[29]) percent body fat, overweight or obese, objectively measured physical activity (GeneActiv© worn by the eldest child only), parent-reported dietary intake for the eldest child (using the Family Nutrition and Physical Activity questionnaire[30]) and behaviour and emotional well-being (using the Strength and Difficulties questionnaire).[31]

To assess the feasibility of collecting outcome data for a future health economic analysis we collected the EQ-5D-5L[32] and ICECAP-A[33] from fathers and the CHU-9D[34-36] from the children.

Progression criteria

Progression criteria were pre-defined to help evaluate whether the feasibility trial should be recommended to progress to a fully powered RCT. These are detailed in Table 4.

Data analysis

For the quantitative analyses, the aim was to assess the progression criteria and feasibility of delivery of a main trial. All analyses were by intention-to-treat and undertaken in Stata V.12. Feasibility outcomes are presented overall and by group with counts, percentages and 95% confidence intervals. Weight change in fathers at 3 and 6-months follow-up are summarised using means, standard deviations and within group 95% confidence intervals. Baseline characteristics are summarised by group and overall and the proposed secondary outcomes of a definitive trial are summarised by group only. Categorical data are presented using counts and percentages, and continuous data are presented using means and standard deviations

For peer review only - http://bmjopen.bmj.com/site/about/guidelines.xhtml

or median and interquartile range where appropriate. Since the study was not powered to detect treatment effects on clinical outcomes, p-values and 95% confidence intervals are not reported.

Audio recordings of qualitative interviews were transcribed verbatim, anonymised and analysed using the framework approach.[37]

Public involvement

A public and patient participation group of eight fathers and two mothers from one of the research sites supported the study from the design stage by contributing to decisions about outcome measures, commenting on intervention materials and participant facing documents, advising on recruitment strategies and dissemination of the findings.

RESULTS

Feasibility of conducting a definitive RCT:

Despite multiple recruitment methods, only 92 men expressed interest in the study, of whom 43 were recruited and randomised (intervention group n=29) (Figure 1). No participants declined randomisation after an assessment visit, nor was randomisation given as a reason for declining after expressing an interest in the study.

Four HDHK-UK courses were delivered, one at site 1 (in a youth centre) and three at site 2 (two in a leisure centre and one in a community centre).

Baseline characteristics of participants

The fathers' mean BMI at baseline was 30.2kg/m^2 (SD 5.1) and their mean age was 40.0 years (range 23.8-56.0). The mean age of their participating children was 7.7 years (range 4.0–11.7). Overall, 60.5% of participants were from minority ethnic groups and 74.4% lived in the two most deprived quintiles of the IMD. Details of the baseline characteristics are presented in Table 1.

For peer review only - http://bmjopen.bmj.com/site/about/guidelines.xhtml

Table 1: Baseline characteristics for fathers and children by treatment arm

	**HDHK-UK programme** (N=29)	**Minimum Intervention** (N=14)	**Overall** (N=43)
**Fathers**			
Age (years)			
Mean (SD), N	39.4 (6.3), 29	41.1 (6.6), 14	40.0 (6.4), 43
Ethnicity			
White British	12 (41.4%)	5 (35.7%)	17 (39.5%)
Non White British	17 (58.6%)	9 (64.3%)	26 (60.5%)
Highest level of qualification			
GCSE, CSE, O level or equivalent	7 (24.1%)	6 (42.9%)	13 (30.2%)
A-level/AS level or equivalent	3 (10.3%)	2 (14.3%)	5 (11.6%)
Degree level or higher	15 (51.7%)	6 (42.9%)	21 (48.8%)
Other	2 (6.9%)	0 (0.0%)	2 (4.7%)
Missing	2 (6.9%)	0 (0.0%)	2 (4.7%)
Legal marital or civil partnership status			
Married or in a registered civil partnership	25 (86.2%)	13 (92.9%)	38 (88.4%)
Divorced or formerly in a civil partnership which is now legally dissolved	1 (3.5%)	0 (0.0%)	1 (2.3%)
Never married and never registered in a civil partnership	1 (3.5%)	1 (7.1%)	2 (4.7%)
Missing	2 (6.9%)	0 (0.0%)	2 (4.7%)
Main spoken language			
English	23 (79.3%)	14 (100.0%)	37 (86.1%)
Urdu	1 (3.5%)	0 (0.0%)	1 (2.3%)
Punjabi	1 (3.5%)	0 (0.0%)	1 (2.3%)
Spanish	1 (3.5%)	0 (0.0%)	1 (2.3%)
Turkish	1 (3.5%)	0 (0.0%)	1 (2.3%)
Missing	2 (6.9%)	0 (0.0%)	2 (4.7%)
Index of Multiple Deprivation quintile, n (%)			
1 (least deprived)	1 (3.5%)	0 (0.0%)	1 (2.3%)
2	1 (3.5%)	1 (7.1%)	2 (4.7%)
3	5 (17.2%)	1 (7.1%)	6 (14.0%)
4	5 (17.2%)	4 (28.6%)	9 (20.9%)
5 (most deprived)	17 (58.6%)	6 (42.9%)	23 (53.5%)
Missing	0 (0.0%)	2 (14.3%)	2 (4.7%)
BMI (kg/m²)			
Mean (SD), N	30.1 (4.8), 29	30.2 (5.8), 14	30.2 (5.1), 43
Weight at baseline (kg)			
Mean (SD), N	90.1 (13.2), 29	92.2 (19.3), 14	

For peer review only - http://bmjopen.bmj.com/site/about/guidelines.xhtml

	**HDHK-UK programme** (N=29)	**Minimum Intervention** (N=14)	**Overall** (N=43)
Percentage body fat (%)			
Mean (SD), N	28.3 (7.6), 28	29.8 (9.0), 14	28.8 (8.0), 42
Waist circumference (cm)			
Mean (SD), N	101.8 (8.5), 19	103.1 (15.0), 14	102.3 (11.5), 33
International physical activity questionnaire[24] (IPAQ-short)			
Low activity	8 (27.6%)	6 (42.9%)	14 (32.6%)
Moderate activity	10 (34.5 %)	4 (28.6%)	14 (32.6%)
Vigorous activity	10 (34.5%)	3 (21.4%)	13 (30.2%)
Missing	1 (3.5%)	1 (7.1%)	2 (4.7%)
Total activity (minutes) from GENEactiv measurement			
Median [IQR]	207.3 [175.8, 270.1]	213.6 [157.4, 248.9]	210.4 [167.7, 264.6]
Missing	4	1	5
Moderate/vigorous activity (minutes) from GENEactiv measurement			
Median [IQR]	110.8 [88.4-141.3]	87.0 [75.9-148.4]	109.7 [83.8-148.4]
Missing	4	1	5
EQ-5D-5L Index score[32] (adult Quality of Life)			
Mean (SD)	0.91 (0.16)	0.95 (0.07)	0.93 (0.13)
Missing	2	0	2
ICECAP-A total capability score[33] (adult wellbeing and Quality of Life)			
Mean (SD)	0.86 (0.15)	0.95 (0.06)	0.89 (0.13)
Missing	1	0	1
**Children**			
	**(N=42)**	**(N=19)**	**(N=61)**
Age (years)			
Mean (SD)	7.7 (2.0)	7.8 (2.2)	7.7 (2.1)
Minimum-Maximum	4.2-11.5	4.0-11.7	4.0-11.7
Sex			
Female	16 (38.1%)	6 (31.6%)	22 (36.1%)
Male	26 (61.9%)	13 (68.4%)	39 (63.9%)
BMI (kg/m²)			
Underweight/healthy	20 (47.6%)	13 (68.4%)	33 (54.1%)
Overweight/obese	16 (38.1%)	4 (21.1%)	20 (32.8%)
Missing	6 (14.3%)	2 (10.5%)	8 (13.1%)
Percentage body fat (%)			
Mean (SD)	24.0 (5.8)	22.9 (4.9)	23.6 (5.5)
Minimum-Maximum	10.3-36.7	16.9-37.3	10.3-37.3

For peer review only - http://bmjopen.bmj.com/site/about/guidelines.xhtml

	**HDHK-UK programme** (N=29)	**Minimum Intervention** (N=14)	**Overall** (N=43)
Missing	6	3	9

Note: All figures presented are N (%) unless otherwise specified.
BMI: body mass index; IQR: interquartile range; SD: standard deviation

Follow-up rates

The follow-up rate at three- and six-months post intervention was 62.8% (n=27); 58.6% (n=17) in the intervention group and 71.4% (n=10) in the control group (figure 1). Participants lost to follow-up at 6-months were more likely to be white British (supplementary web-table 2).

Level of completion of follow-up questionnaires

Researchers experienced challenges in arranging appointments for data collection. Despite repeated reminders, researchers often arrived at a family's house to find they were no longer available. Work and after-school activities made finding times for recruitment visits difficult and impacted recruitment numbers. For fathers and their children who completed data collection appointments, the processes worked well. In the main, the completeness of the data was acceptable with the exception of high levels of missing data for the IPAQ questionnaire and fathers' waist circumference, which was frequently refused (23% missing at baseline).

Fathers' weight change in the intervention group

Table 2 summarises fathers' weight change from baseline. The intervention group had a 2.9kg (95% CI 0.6 to 5.1) reduction in weight at 6-months follow-up.

For peer review only

Table 2 Weight change and secondary outcome measures for fathers by treatment arm

	3-months		6-months	
	**HDHK UK programme** (N=29)	**Minimum Intervention** (N=14)	**HDHK UK programme** (N=29)	**Minimum Intervention** (N=14)
**Weight change from baseline (kgs)**				
Mean (SD), N [95% CI]	-1.8 (2.5), 17 [-3.1 to -0.5]	-1.2 (3.3), 9 [-3.7 to 1.3]	-2.9 (4.1), 15 [-5.1 to -0.6]	-2.0 (3.6), 10 [-4.6 to 0.6]
**Change from baseline in waist circumference (cm)**				
Mean [SD], N	-10.8 (18.0), 9	3.4 (9.3), 6	-5.2 (5.0), 6	-2.8 (6.4), 5
**Change from baseline in % body fat**				
Mean [SD], N	-1.5 (3.1), 16	-0.3 (2.6), 9	-2.2 (3.2), 14	-2.3 (3.9), 9
**Physical activity measured by a GENEactive accelerometer**				
Median for total activity (minutes) [IQR], N	208.8 [185.6, 287.0], 17	168.3 [147.8, 194.0], 9	239.6 [194.3, 287.0], 11	146.2 [125.0, 230.6], 9
Median for moderate/vigorous activity [IQR], N	113.3 [99.3, 151.3], 17	84.2 [69.4, 106.2], 9	125.6 [101.3, 163.1], 11	68.2 [59.1, 86.5], 9
**EQ-5D-5L**[32] (adult health related quality of Life)				
Mean [SD], N	0.87 (0.19), 17	0.94 (0.10), 9	0.92 (0.14), 14	0.94 (0.13), 9
**ICECAP-A**[33] (adult capability and wellbeing)				
Mean [SD], N	0.92 (0.09), 17	0.95 (0.05), 9	0.89 (0.13), 14	0.92 (0.07), 9
**Lost ≥5% body mass**				
Yes	2 (6.9%)	1 (7.1%)	9 (31.0%)	1 (7.1%)
No	15 (51.7%)	8 (57.1%)	6 (20.7%)	9 (64.3%)
Missing	12 (41.4%)	5 (35.7%)	14 (48.3%)	4 (28.6%)
**Parenting for physical activity**				

For peer review only - http://bmjopen.bmj.com/site/about/guidelines.xhtml

Activity Support scale for Multiple Groups (ACTS-MG)[27]				
Mean (SD), N	15.6 (3.5), 17	13.0 (2.3), 9	16.4 (2.2), 14	13.7 (1.5), 9
Parenting strategies for Eating and Activity Scale[26] (Mean (SD), N)				
Limit setting	8.4 (1.9), 17	8.8 (1.4), 9	7.5 (2.1), 13	9.1 (1.2), 9
Control	2.7 (1.6), 17	2.1 (1.3), 9	3.4 (1.3), 13	2.6 (1.7), 9
Monitoring	7.3 (1.5), 17	7.4 (1.1), 9	7.4 (1.5), 14	7.7 (0.7), 9
Disciplining	5.2 (2.3), 17	4.7 (2.4), 9	5.2 (2.7), 14	6.3 (2.2), 9
Co-physical activity	3.5 (0.9), 17	2.7 (0.7), 9	3.8 (1.3), 14	3.2 (0.8), 9
**Father-child relationship[25]** (Mean (SD), N)				
[a]Disciplinary warmth	22.9 (4.0), 16	23.0 (1.6), 9	23.8 (3.6), 14	23.3 (3.0), 9
[b]Personal relationships	31.4 (5.0), 16	28.9 (2.6), 9	30.6 (4.8), 14	30.0 (2.9), 9

BMI: body mass index; EQ-5D-5L: EuroQol; ICECAP-A: ICEpop CAPability measure for Adults; IQR: interquartile range; SD: standard deviation;

[a]Disciplinary warmth= praise + shared decision making + rationale; [b]Personal relationships (pro-social + intimacy + nurturance + companionship)

For peer review only

Secondary outcomes

Table 2 summarises other secondary outcomes for fathers and Table 3 for the children. The intervention group fathers had favourable reduction in waist circumference; 31% (n=9) achieved a 5% reduction in body mass at 6-months. There were no serious adverse events requiring hospital admission, or adverse events requiring medical attention during the intervention.

For peer review only

For peer review only - http://bmjopen.bmj.com/site/about/guidelines.xhtml

Table 3: Secondary outcome measures for eldest child by treatment arm

	3-months		6-months	
	**HDHK UK programme** (N=29)	**Minimum Intervention** (N=14)	**HDHK UK programme** (N=29)	**Minimum Intervention** (N=14)
**Change from baseline in BMI z-score**				
Mean [SD], N	-0.131 (0.272), 14	0.016 (0.346), 8	-0.016 (0.299), 12	0.039 (0.449) ,10
**Change from baseline in % body fat**				
Mean [SD], N	-0.58 (1.17), 13	0.04 (2.84), 8	-0.80 (1.48), 11	-0.88 (3.07), 8
**Categorised as overweight or obese (%)**				
Underweight/healthy	8 (27.6%)	6 (42.9%)	7 (24.1%)	6 (42.9%)
Overweight/obese	7 (24.1%)	3 (21.4%)	6 (20.7%)	4 (28.6%)
Missing	14 (48.3%)	5 (35.7%)	16 (55.2%)	4 (28.6%)
**Physical activity measured by a GENEactive accelerometer**				
Median for total activity (minutes) [IQR], N	342.3 [262.7, 427.7], 17	277.0 [272.5, 314.3], 9	347.3 [321.6, 384.0], 10	312.8 [245.4, 456.8], 8
Median for moderate/vigorous activity [IQR], N	73.5 [34.7, 99.3], 17	57.0 [26.8, 73.0], 9	73.2 [49.0, 105.7], 10	56.0 [32.4, 110.6], 8
**Family nutrition & physical activity questionnaire[30]**				
Mean [SD], N	62.3 (7.7), 16	61.1 (5.2), 9	61.2 (6.5), 9	62.1 (3.0), 8
**CHU-9D[34-36]** (child utility measure)				
Mean [SD], N	0.89 (0.09), 15	0.93 (0.04), 9	0.92 (0.09), 11	0.92 (0.11), 8
**[a]Strengths and difficulties questionnaires[31] (Total SDQ)**				
Mean [SD],N	-	-	7.4 (3.8), 12	11.2 (6.1), 6

[a]SDQ: Each 1 point increase in the total difficulties score corresponds with an *increase in the risk* of developing a mental health disorder.

BMI: body mass index; CHU-9D: Child Health Utility-9D

For peer review only - http://bmjopen.bmj.com/site/about/guidelines.xhtml

Feasibility and acceptability of programme delivery

Ability to recruit and retain facilitators

Site 1 provided health trainers employed by the local authority for the fathers' education component and an independent fitness trainer delivered the physical activity elements. At site 2, two courses were delivered in a leisure centre by their staff and one course was delivered in a community centre by sports coaches employed by a coaching organisation.

We experienced challenges in delivering training due to loss of potential facilitators as a result of rapid turnover of staff from the various delivery organisations. Three forms of training were delivered: a two-day HDHK training workshop delivered by the Fatherhood Institute, who had previously been trained by the Australian team; a half day of top-up training by the Fatherhood Institute; and more flexible training for facilitators recruited after these sessions, delivered by a member of the research team (TG) who had previous experience of coaching training.

We intended that two courses would be delivered at each site. However, due to organisational restructuring, the health trainers were only able to deliver one course.

Challenges to recruiting participants

There were significant challenges with recruitment. We obtained permission from 40 organisations to recruit on their premises (including 11 schools, 7 faith organisations, 6 sport/leisure centres), but it was a time consuming process. Communication with schools was slow and recruitment via children and 'mothers', was not a successful strategy. Fathers were hard to engage during recruitment activities and session timings were often not convenient for their family. Additionally, as a result of a change in sites after funding was awarded (due to a local site withdrawing funding for adult weight management services), the sites involved considerable researcher travel time.

Deliver sessions at a time and location convenient for participants

Aligning venue, facilitator and participant availability was a major challenge and resulted in considerable delays in commencing programme delivery. Evening sessions were difficult due to fathers' work commitments, and children's meal and bed times. Although weekends were reported to be convenient by participants, the availability of facilitators and community venues were more limited.

> *I wasn't finishing work until 5 o'clock and then I was having to fight my way through the traffic to get there. If it had been any later, it's harder for the kids then because they've*

For peer review only - http://bmjopen.bmj.com/site/about/guidelines.xhtml

got to get up for school the next week. ... and weekends wouldn't be any good because the leisure centre would be packed. (ID B-008)

Session timings were also the main reason given by participants for non-attendance at intervention sessions.

It started at half past five which is obviously rush hour time. Dads are leaving work ... In the end, what happened was it went from a group of six dads and their children to the last session being just three dads …. I think that wasn't due to their motivation but their work time commitments. (ID B-068)

Fidelity of delivery

The intervention was delivered with high fidelity. The style of delivery and the small group sizes meant group discussions were encouraged; however, facilitators reported finding it challenging to deliver all the session content in the allocated time. Researchers sometimes observed some session content being skipped or a delay in the start of the subsequent practical session. Despite the challenges, the facilitator's ensured key content was delivered, which was verified in participant interviews where they spoke of the key messages the course had focused on.

Acceptability of adapted intervention to participants

Twenty participants (69%) attended an intervention course at least once; 15 attended (52%) at least five sessions. Fathers who attended the sessions and took part in a qualitative interview were positive about their experiences. Feedback sheets, observations and qualitative interviews with both participants and facilitators consistently showed high levels of acceptability towards the intervention programme.

It [HDHK-UK programme] was brilliant overall. I really enjoyed it. The kids enjoyed it. (ID B-068)

The group sessions were appreciated by participants:

I enjoyed the group-based elements … it allowed to people to bounce off, talk about - and also the blokes, a little bit of competitive edge, especially when the weight round was coming. So I think it benefited me and I preferred the group format rather than just individually between me and my children. (ID A-077)

For peer review only - http://bmjopen.bmj.com/site/about/guidelines.xhtml

A strong theme was the appreciation of time spent with their children "*I'm working during the week and it's just nice to have that dad-and-daughter time when just for a couple of hours it was just us and I think we've really benefited from that"* (ID A-072)'

Participants' spoke highly of the facilitators for the education sessions; *'Fantastic, very well presented, well engaging, knowledgeable.* (ID A-058)' and the physical activity sessions: '*they were excellent, really good lads…we were always busy, always sweating, it was all good'* (ID B-089). Similarly, the facilitators spoke positively of the programme.

A key challenge was delivery over the UK winter:

> *The weather had been against us a lot of the times, I think if we had done it now in the summer, I think it would have been a lot better for us (ID B-089)*

> *…although we've been out Saturdays and Sundays it's trying to fit it into darker nights when it's been cold and it's been wet or it's been snowing or whatever else, that's been difficult. (*ID A-072)

Progression criteria

Progression criteria for a full trial (Table 4) were met for intervention acceptability to participants who attended and fidelity of delivery, recruitment to the trial was low and attrition was high (37.2%, n=16). Mean weight loss in the intervention arm was marginally below the pre-specified ≥3kg in the participants who were followed-up.

For peer review only - http://bmjopen.bmj.com/site/about/guidelines.xhtml

Table 4 Progression criteria for a full trial

Progression criterion	Assessment
Intervention is acceptable to a majority of fathers and families from differing BAME and socio-economic backgrounds;	Achieved
Randomisation occurs and more than 80 per cent of those assessed accept randomisation;	Achieved
Recruitment of at least 68 out of the planned 90 fathers (75 per cent) within the 4-month time frame;	43 (48%)
Intervention implemented with fidelity in 75 per cent of observations (see 'process evaluation' section above);	Achieved
Attendance 70 per cent attending at least 5/9 of the planned sessions;	15 (52%)
More than 70 per cent follow up at three and six-months;	27 (62.8%)
Mean weight loss in the intervention arm of ≥3kg.	2.9kg (95% CI -5.1, -0.6)

DISCUSSION

The aims of the current study were to assess the feasibility of delivering a culturally adapted HDHK programme in a socioeconomically deprived UK setting, and the feasibility of conducting a definitive RCT. Overall, it was possible to recruit and train facilitators to deliver the intervention programme with high fidelity. The programme was rated highly by the attending participants and delivery teams and those who participated achieved weight loss comparable with other studies.[13, 16] However, recruitment of participants was difficult, and aligning participant, venue and facilitator availability to deliver the programme was a major challenge. As such, participant recruitment and attendance rates were low and the progression criteria for the study were not met.

Despite multiple strategies, encouraging fathers to engage with the study was challenging. Attempts to enhance recruitment through schools, an approach used in the Australian setting, proved more difficult than anticipated. Schools are often involved in multiple projects and programmes, which may have meant they felt they did not have added capacity for HDHK-UK. It was recognised during recruitment that there can be sensitivities around discussing weight. To try to offset this, the opportunity for fathers to spend time with their children was emphasised. Challenges in recruitment have also been recognised in a similar study with fathers[38] and as a common theme in a systematic review of weight loss trials in men.[13, 14] However, the findings and experiences from this current study differ from those of the

For peer review only - http://bmjopen.bmj.com/site/about/guidelines.xhtml

Australian HDHK RCT.[16, 17] Notably, the Australian study used a wait-list study design where all families were guaranteed to receive the programme at some point, which may have been more appealing for participants. An additional difference was a greater availability of sessions.

Nearly a third of participants allocated to the intervention group did not engage with the programme. This has been seen in other group based health related programmes[39, 40,41] but differs from the HDHK trial in Australia.[16, 17] The primary reason given for non-attendance or non-completion of the programme was that the session timings were not suitable, which was corroborated by the research team who found identifying a convenient time for programme delivery to be one of the most significant challenges of the study. Evening sessions had to align to fathers working patterns and children's school and bed-time routines, resulting in sessions often being set when traffic was at its heaviest. This was compounded by running the sessions in the winter with dark nights and poor weather. Weekend sessions often clashed with family activities and venue and facilitator availability were more limited. Poor weather is likely to have also impacted recruitment, especially at schools where parents were not keen to stop to talk to the study team in rainy or cold conditions. Delivery in summer and offering more choice of sessions might mitigate some of these challenges.

The facilitators reported it to be challenging to deliver the full content of the father's education session within the allocated 30 minutes, exacerbated by participants arriving late. The approach to sessions was to encourage interaction and discussion, which was appreciated by participants, and necessary if the group size was small. However, this at times challenged the fidelity of delivery and if the programme were to be rolled out we would recommend reducing session content further.

The youngest children participating in the current study had difficulty in engaging with written materials; four year olds were not eligible in the Australian studies.[16, 17, 41]

Strengths and Limitations

The study has a number of strengths. Whilst successful weight management trials in men have been reported[10, 12, 13, 16, 17] most participants in weight loss trials are women.[42, 43] In addition, very few specifically target ethnic minority population groups[42] or recruit from socio-economically disadvantaged populations, which is a unique contribution of this study.

This study also had limitations. First, the small number of participants recruited and attending the programme meant some adaptation for a smaller group size was required, but meant some of the group atmosphere was reduced. Those who attended enjoyed the programme, and whilst the remainder advised that session timing was their main reason for non-attendance,

For peer review only - http://bmjopen.bmj.com/site/about/guidelines.xhtml

the small sample size creates bias, especially for the qualitative interviews. Whilst every attempt was made to interview non-attendees and non-completers, engagement was low. We had also planned to interview participants with their partners and children present, but logistically this proved difficult to arrange; many of the children were too young to participate in an interview and most fathers opted for a phone interview as they found this more convenient.

Another limitation was the change in facilitation staff throughout the study, resulting in the need to run individualised training sessions after the original training delivered by the Fatherhood Institute. Whilst the additional training was delivered by an experienced researcher (TG), the process presented a significant time commitment and may have led to differences in delivery style.

Due to the study timeline and working with school terms and avoiding Ramadan, the intervention was delivered at the height of winter, a contrast to the Australian programme which is delivered only in summer months. This is likely to have impacted both recruitment and intervention attendance.

The session observations, and feedback from participants and facilitators were overwhelmingly positive. Despite acknowledged limitations, the adapted HDHK programme was delivered and received well. The observed outcome measure of weight change across the programme was promising and the data collected showed good levels of completion.

Future research

At this time the Trial Steering Committee did not recommend progressing to a full RCT in the context in which this study was set. However, there may be other settings within the UK in which some of the barriers that were faced would either not occur, or could be addressed or ameliorated. In line with the recommendations from the study PPI group, delivery of the programme outside the context of weight management may result in higher recruitment.

CONCLUSIONS

The majority of difficulties experienced in this study stem from the setting and context under which it was to be delivered, and whilst we do not recommend progressing this feasibility study to a full RCT, we recognise that the outcomes and recommendations made may have been different had the study been trialled in a different setting. A number of challenging circumstances converged within the study which likely compounded further the difficulties encountered in delivery: recruitment difficulties, the untimely change in facilitation staff and the poor weather throughout recruitment and delivery. The intervention was rated highly by

For peer review only - http://bmjopen.bmj.com/site/about/guidelines.xhtml

those who attended and the weight loss achieved by the intervention participants was promising, although no definitive conclusions can be drawn owing to the small sample size.

Author contribution

KJ, PA, AB, CC, AD, EF, LJ, MP, MY and PM conceived the study; KJ was PI; YS undertook the statistical analysis, PH was senior statistician; TG was study co-ordinator and led the process evaluation; LJ was the qualitative lead; TG and KH undertook the qualitative interviews and analysis; PM and CC conceived and designed the original HDHK intervention; PM and MY advised on training and delivery; AE was PPI lead; all authors interpreted the findings; TG and KJ drafted the manuscript; all authors and commented on paper drafts and agreed the final version.

Competing interests

Professors Philip Morgan and Clare Collins designed the original Healthy Dads, Healthy Kids Programme in Australia. The other authors declare no competing interests.

Acknowledgements

We would like to thank all participating families, the local authorities for their support and direction and the facilitators who delivered the programme. We acknowledge members of the study steering committee: Professor John Wright, Bradford Institute for Health Research (Chair); Professor Pat Hoddinott, University of Stirling; Elaine Nicholls, Keele University; Mr Ray Fiveash, PPI representative. We acknowledge Kathy Jones and Cassius Campbell who delivered the training programme and Jeremy Davies from the Fatherhood Institute. We thank Jeszemma Garratt from the Fatherhood Institute and the Parent Advisory Panel and staff who contributed to the research delivery: Dr Khaled Ahmed, Felicity Brant, David Sardar and Meanaz Akhtar. We acknowledge academics from the University Newcastle, Australia who developed the original Healthy Dads, Healthy Kids programme and who supported the study through the development of training materials and shared their experience of delivery in the Australian setting – Professor David Lubans and Kristen Saunders.

Funding

Study funding was granted in October 2015 by the National Institute of Health Research Public Health Research programme (Ref 14/185/13); KJ is part-funded by NIHR Collaborations for Leadership and Health Research and Care West Midlands. The views expressed are those of the authors and not necessarily those of the NHS, the NIHR, or the Department of Health and Social Care.

For peer review only - http://bmjopen.bmj.com/site/about/guidelines.xhtml

Trial registration

ISRCTN registry January 2017:

(ISRCTN16724454.https://doi.org/10.1186/ISRCTN16724454).

Ethical approval

Ethical approval for the two phases of the study was obtained from The University of Birmingham Science, Technology, Engineering and Mathematics Ethical Review Committee (16th January 2017 (ethics reference; ERN_16-1323).

Data sharing: All data are available on request from the corresponding author

For peer review only

REFERENCES

1. *The NHS Long Term Plan* London: NHS; 2019.

2. Kopelman P. Health risks associated with overweight and obesity. *Obes Rev* 2007;**8 Suppl 1**:13-7. http://dx.doi.org/10.1111/j.1467-789X.2007.00311.x

3. Prospective Studies Collaboration. Body-mass index and cause-specific mortality in 900 000 adults: collaborative analyses of 57 prospective studies. *The Lancet* 2009;**373**:1083-96. http://dx.doi.org/https://doi.org/10.1016/S0140-6736(09)60318-4

4. Public Health England. *Health Matters: Obesity and the food environment.* Public Health England; 2017. URL: https://www.gov.uk/government/publications/health-matters-obesity-and-the-food-environment/health-matters-obesity-and-the-food-environment--2 (Accessed 28/05/2018).

5. National Statistics. *Statistics on Obesity, Physical Activity and Diet. England 2017.* . National Statistics; 2017. URL: https://files.digital.nhs.uk/publicationimport/pub23xxx/pub23742/obes-phys-acti-diet-eng-2017-rep.pdf. (Accessed 28/05/2018).

6. NHS Digital. *Health Survey for England 2016. Adult overweight and obesity.* . NHS Digital; 2016. URL: https://digital.nhs.uk/data-and-information/publications/statistical/health-survey-for-england/health-survey-for-england-2016 (Accessed 28/05/2018).

7. National Institute for Health and Clinical Excellence. *BMI: preventing ill health and premature death in black, Asian and other minority ethnic groups. Public health guideline [PH46]*; 2013.

8. Gettler LT, Sarma MS, Gengo RG, Oka RC, McKenna JJ. Adiposity, CVD risk factors and testosterone: Variation by partnering status and residence with children in US men. *Evolution, Medicine, and Public Health* 2017;**2017**:67-80. http://dx.doi.org/10.1093/emph/eox005

9. Garfield CF, Duncan G, Gutina A, Rutsohn J, McDade TW, Adam EK*, et al.* Longitudinal Study of Body Mass Index in Young Males and the Transition to Fatherhood. *Am J Mens Health* 2016;**10**:Np158-np67. http://dx.doi.org/10.1177/1557988315596224

10. Morgan PJ, Callister R, Collins CE, Plotnikoff RC, Young MD, Berry N*, et al.* The SHED-IT community trial: a randomized controlled trial of internet- and paper-based weight

For peer review only - http://bmjopen.bmj.com/site/about/guidelines.xhtml

loss programs tailored for overweight and obese men. *Ann Behav Med* 2013;**45**:139-52. http://dx.doi.org/10.1007/s12160-012-9424-z

11. Morgan PJ, Collins CE, Plotnikoff RC, Cook AT, Berthon B, Mitchell S*, et al.* The impact of a workplace-based weight loss program on work-related outcomes in overweight male shift workers. *J Occup Environ Med* 2012;**54**:122-7. http://dx.doi.org/10.1097/JOM.0b013e31824329ab

12. Hunt K, Wyke S, Gray CM, Anderson AS, Brady A, Bunn C*, et al.* A gender-sensitised weight loss and healthy living programme for overweight and obese men delivered by Scottish Premier League football clubs (FFIT): a pragmatic randomised controlled trial. *Lancet* 2014;**383**:1211-21. http://dx.doi.org/10.1016/s0140-6736(13)62420-4

13. Robertson C, Archibald D, Avenell A, Douglas F, Hoddinott P, van Teijlingen E*, et al.* Systematic reviews of and integrated report on the quantitative, qualitative and economic evidence base for the management of obesity in men. *Health Technol Assess* 2014;**18**:v-vi, xxiii-xxix, 1-424. http://dx.doi.org/10.3310/hta18350

14. Young MD, Morgan PJ, Plotnikoff RC, Callister R, Collins CE. Effectiveness of male-only weight loss and weight loss maintenance interventions: a systematic review with meta-analysis. *Obes Rev* 2012;**13**:393-408. http://dx.doi.org/10.1111/j.1467-789X.2011.00967.x

15. Morgan PJ, Young MD, Lloyd AB, Wang ML, Eather N, Miller A*, et al.* Involvement of Fathers in Pediatric Obesity Treatment and Prevention Trials: A Systematic Review. *Pediatrics* 2017;**139**:e20162635. http://dx.doi.org/10.1542/peds.2016-2635

16. Morgan PJ, Collins CE, Plotnikoff RC, Callister R, Burrows T, Fletcher R*, et al.* The 'Healthy Dads, Healthy Kids' community randomized controlled trial: a community-based healthy lifestyle program for fathers and their children. *Prev Med* 2014;**61**:90-9. http://dx.doi.org/10.1016/j.ypmed.2013.12.019

17. Morgan PJ, Lubans DR, Callister R, Okely AD, Burrows TL, Fletcher R*, et al.* The 'Healthy Dads, Healthy Kids' randomized controlled trial: efficacy of a healthy lifestyle program for overweight fathers and their children. *Int J Obes (Lond)* 2011;**35**:436-47. http://dx.doi.org/10.1038/ijo.2010.151

18. Jolly K, Griffin T, Sidhu M, Adab A, Burgess A, Collins CE*, et al.* A weight management programme for fathers of children aged 4-11: cultural adaptation and the Healthy Dads, Healthy Kids UK feasibility RCT. *Public Health Research* in press.

19. Office for National Statistics. *Local Area Report for areas in England and Wales.* . 2017. URL: https://www.nomisweb.co.uk/reports/localarea (Accessed 09/10/2018).

20. Golan M, Weizman A. Familial approach to the treatment of childhood obesity: conceptual mode. *J Nutr Educ* 2001;**33**:102-7.

21. Golan M, Weizman A, Apter A, Fainaru M. Parents as the exclusive agents of change in the treatment of childhood obesity. *Am J Clin Nutr* 1998;**67**:1130-5. http://dx.doi.org/10.1093/ajcn/67.6.1130

22. Bandura A, & National Inst of Mental Health. *Prentice-Hall series in social learning theory. Social foundations of thought and action: A social cognitive theory.* . Englewood Cliffs, NJ, US: Prentice-Hall, Inc.; 1986.

23. Department for Communities and Local Government. *The English Indices of Deprivation 2015*. National Statistics; 2015. URL: https://assets.publishing.service.gov.uk/government/uploads/system/uploads/attachment_data/file/465791/English_Indices_of_Deprivation_2015_-_Statistical_Release.pdf (Accessed 09/10/2018).

For peer review only - http://bmjopen.bmj.com/site/about/guidelines.xhtml

24. Craig CL, Marshall AL, Sjostrom M, Bauman AE, Booth ML, Ainsworth BE*, et al.* International physical activity questionnaire: 12-country reliability and validity. *Med Sci Sports Exerc* 2003;**35**:1381-95. http://dx.doi.org/10.1249/01.mss.0000078924.61453.fb

25. Furman W, Giberson R. Identifying the links between parents and their children's sibling relationships. In: Shulman S, editor. *Human development, Vol. 7. Close relationships and socioemotional development*Westport, CT, US: Ablex Publishing.; 1995:95-108.

26. Larios SE, Ayala GX, Arredondo EM, Baquero B, Elder JP. Development and validation of a scale to measure Latino parenting strategies related to children's obesigenic behaviors. The parenting strategies for eating and activity scale (PEAS). *Appetite* 2009;**52**:166-72. http://dx.doi.org/10.1016/j.appet.2008.09.011

27. Lampard AM, Nishi A, Baskin ML, Carson TL, Davison KK. The Activity Support Scale for Multiple Groups (ACTS-MG): Child-reported physical activity parenting in African American and non-Hispanic White families. *Behavioral medicine (Washington, DC)* 2016;**42**:112-9. http://dx.doi.org/10.1080/08964289.2014.979757

28. Pan H, Cole T. LMS growth, a Microsoft Excel add-in to access growth references based on the LMS methods. In. 2.77 ed; 2012.

29. McCarthy HD, Jarrett KV, Crawley HF. The development of waist circumference percentiles in British children aged 5.0-16.9 y. *Eur J Clin Nutr* 2001;**55**:902-7. http://dx.doi.org/10.1038/sj.ejcn.1601240

30. Ihmels MA, Welk GJ, Eisenmann JC, Nusser SM. Development and preliminary validation of a Family Nutrition and Physical Activity (FNPA) screening tool. *The International Journal of Behavioral Nutrition and Physical Activity* 2009;**6**:14-. http://dx.doi.org/10.1186/1479-5868-6-14

31. Goodman R. Psychometric Properties of the Strengths and Difficulties Questionnaire. *Journal of the American Academy of Child & Adolescent Psychiatry* 2001;**40**:1337-45. http://dx.doi.org/10.1097/00004583-200111000-00015

32. Herdman M, Gudex C, Lloyd A, Janssen MF, Kind P, Parkin D*, et al.* Development and preliminary testing of the new five-level version of EQ-5D (EQ-5D-5L). *Quality of Life Research* 2011;**20**:1727-36. http://dx.doi.org/10.1007/s11136-011-9903-x

33. Al-Janabi H, N Flynn T, Coast J. Development of a self-report measure of capability wellbeing for adults: the ICECAP-A. *Quality of Life Research* 2012;**21**:167-76. http://dx.doi.org/10.1007/s11136-011-9927-2

34. Stevens K. Developing a descriptive system for a new preference-based measure of health-related quality of life for children. *Quality of Life Research* 2009;**18**:1105-13. http://dx.doi.org/10.1007/s11136-009-9524-9

35. Stevens K. Working with children to develop dimensions for a preference-based, generic, pediatric, health-related quality-of-life measure. *Qualitative Health Research* 2010;**20**:340-51.

36. Stevens K. Assessing the performance of a new generic measure of health-related quality of life for children and refining it for use in health state valuation. *Applied Health Economics and Health Policy* 2011;**9**:157-69. http://dx.doi.org/10.2165/11587350-000000000-00000

37. Gale NK, Heath G, Cameron E, Rashid S, Redwood S. Using the framework method for the analysis of qualitative data in multi-disciplinary health research. *BMC Medical Research Methodology* 2013;**13**:117-. http://dx.doi.org/10.1186/1471-2288-13-117

38. Mobley A, Gans K, Adamsons K, Huedo-Medina T. Healthy Fathers, Healthy Kids: Feasibility of a Father-focused Childhood Obesity Prevention Program for Low-income

For peer review only

For peer review only - http://bmjopen.bmj.com/site/about/guidelines.xhtml

Families with Preschool Aged Children (OR13-03-19). *Current developments in nutrition* 2019;**3**:nzz050.OR13-03-19. http://dx.doi.org/10.1093/cdn/nzz050.OR13-03-19

39. Jolly K, Lewis A, Beach J, Denley J, Adab P, Deeks JJ*, et al.* Comparison of range of commercial or primary care led weight reduction programmes with minimal intervention control for weight loss in obesity: Lighten Up randomised controlled trial. *BMJ* 2011;**343**. http://dx.doi.org/10.1136/bmj.d6500

40. British Heart Foundation. *The National Audit of Cardiac Rehabilitation (NACR) annual statistical report 2017*; 2018.

41. Pallan M, Griffin, T., Hurley, K.L., Lancashire, E., Blissett, J., Frew, E., Griffith, L., Hemming, K., Jolly, K., McGee, E., Thompson, JL., Jackson, L., Gill, P., Parry, J., Adab, P. Cultural adaptation of an existing children's weight management programme: the CHANGE intervention development and feasibility RCT. . *Health Technology Assessment* 2018.

42. Pagoto SL, Schneider KL, Oleski JL, Luciani JM, Bodenlos JS, Whited MC. Male Inclusion in Randomized Controlled Trials of Lifestyle Weight Loss Interventions. *Obesity* 2012;**20**:1234-9. http://dx.doi.org/doi:10.1038/oby.2011.140

43. NICE. *Weight management: lifestyle services for overweight or obese adults: Public Health Guidleine [PH53]*; 2014.

For peer review only

For peer review only - http://bmjopen.bmj.com/site/about/guidelines.xhtml

Figure 1: Participant Flow through HDHK-UK for fathers

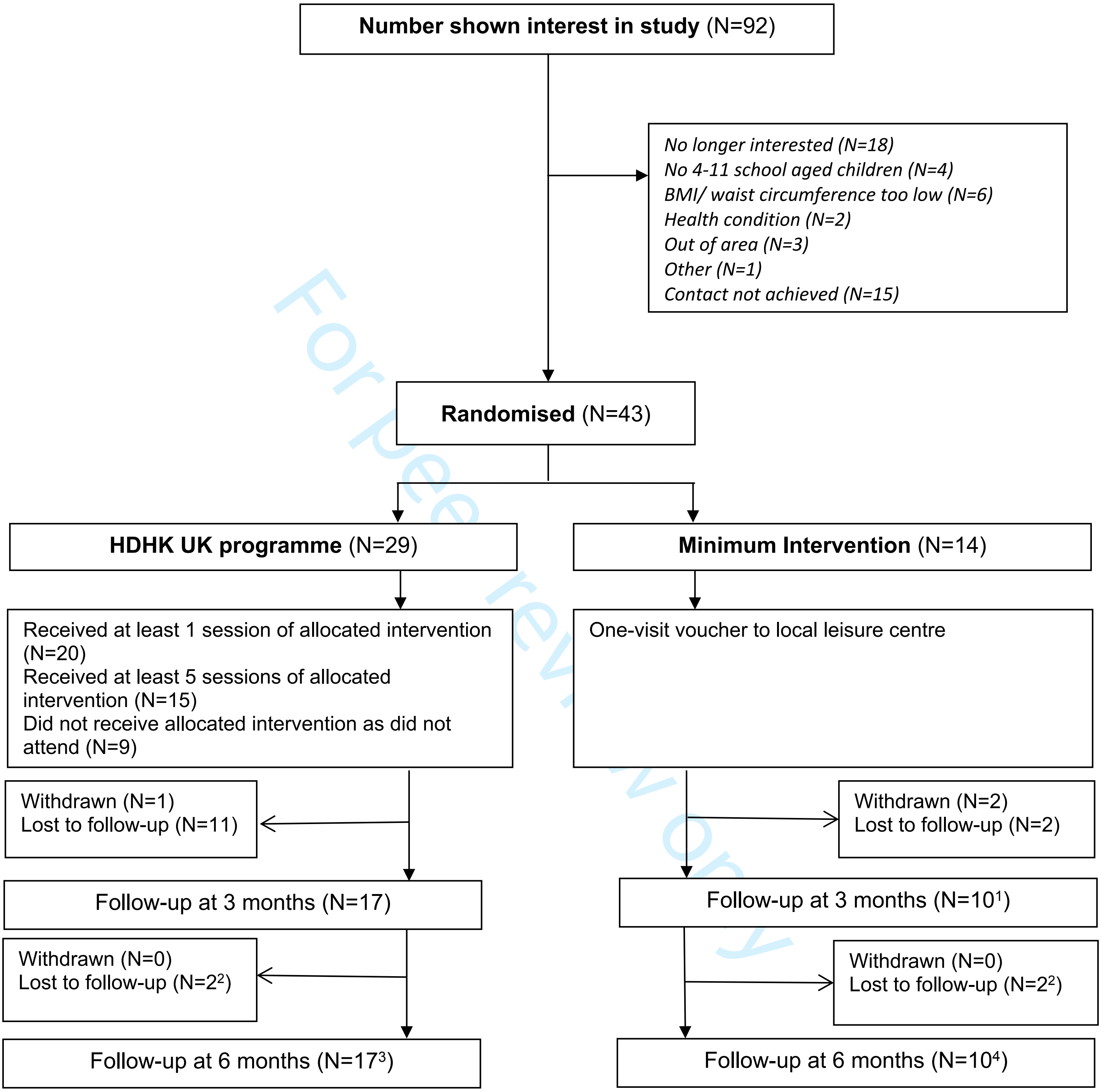

[1] One participant completed questionnaire only;
[2] Lost to follow up out of those participants who completed follow up at month 3;
[3] 15 participants continued from the previous 17 who completed the follow up at month 3; 2 participants returned from lost to follow up at month 3;
[4] 8 participants continued from the 10 who completed follow up at month 3, 2 participants returned from lost to follow up at month 3.

For peer review only

Web-table 1: The HDHK UK programme reported according to the Template for Intervention Description and Replication (TIDieR) checklist

**Item**	**Item description**
1. Name	Healthy Dads, Health Kids UK
2. Why	Following the success of the Healthy Dads, Healthy Kids programme in Australia the aim of adapting the programme was to test the suitability of the programme to an ethnically diverse UK population.<br><br>The primary aim of adapting the HDHK programme was to increase acceptability of the programme to a socio-economically disadvantaged, ethnically diverse UK population.
3 & 4: Materials and Procedures	Fathers of primary school aged children who were classed as overweight or obese (BMI >25kg/m^2 (>23kg/m^2 for fathers of South Asian origin) with primary school aged children (4-11 years) were invited to take part in the HDHK study. Participants randomised to the intervention group were asked to attend the HDHK intervention programme with their children. The intervention programme involved a 90 minute session once a week for nine weeks with weekly goals and activities to be completed in their own time<br><br>**<u>Materials provided to the participants</u>**<br><br>Fathers and children were provided with an intervention pack:<br><br>**Fathers:**<br><br>- A handbook to accompany the father-only education sessions<br>- A logbook for monitoring of weekly activities including step counter, weight chart etc.<br>- A HDHK branded black T-shirt<br>- Nine 'Green slips' - cards with weekly activities to be completed either on their own, with their children or as a family<br>- A certificate (presented by their children) on completion of the programme<br><br>**Children:**<br><br>- A workbook to work through as part of the children-only education sessions. This included weekly activities to complete with their father<br>- Activity cards with instructions how to practice fundamental movement skills and a spinner (as part of a game to select which card to pick each day)<br>- A HDHK branded green T-shirt<br>- A certificate (presented by facilitators) on completion of the programme<br><br>**<u>Materials provided to the facilitators</u>**<br><br>- HDHK branded red T-shirt<br>- Facilitators guide to intervention delivery

For peer review only - http://bmjopen.bmj.com/site/about/guidelines.xhtml

	- Father-only education session facilitators were provided with the PowerPoint slides for delivery<br>- Access to on-line demonstrations of the physical activity components of the programme<br>- Exercise<br><br>Each week the 90 minute course followed the same format:<br><br>**0-15 minutes. Father and children review of their weekly goals with facilitators**<br><br>- A review session where participants and their children updated the facilitators on their activity and if they had completed their weekly tasks the children were given a sticker for their workbooks<br>- This section also included a father-child bonding activity where they were encouraged to talk to each other or play a short game such as 'thumb war' or guess each other's favourite movie/food/activity.<br>- Fathers had opportunity to use scales to weigh themselves and complete their log book<br><br>**15-45 minutes. Fathers ('father-only') and children ('children-only') participate in separate education sessions**<br><br>- Father only sessions were delivered by the facilitator using a pre-prepared PowerPoint slide show which was delivered encouraging discussion around the key points. There were also additional activities each week to be completed in the logbook such as setting goals (weight, physical activity of nutrition goals), calculating daily energy needs,<br>- Every week fathers were asked to report on their weight and average number of daily steps (aiming to 10,000 steps/day)<br>- Child-only education sessions were delivered by working through the children's workbook. The session content followed that of the father sessions but made simple to be child appropriate.<br>- Children were also asked to select two activities each week to complete with their fathers. The activities were planned around physical activity or nutrition, such as 'how many press ups can Dad do in 1minute', 'set up an obstacle course', 'get Dad to drink at least 8 glasses of water a day'.<br><br><u>Fathers' sessions:</u><br><br>Week 1: Dads matter in children's health - Highlights the unique influence of dads in contributing to the physical and mental health of children.<br><br>Week 2: Weight management for men - Explores the challenges of healthy eating in the modern world, outlines the mathematics of weight loss and setting SMART goals to achieve activity and dietary ambitions.<br><br>Week 3: Being a healthy dad – Strategies to enhance your and your family's life. Highlights 9 weight loss tips for men, tells dads

For peer review only

	how to 'stay on track' and provides advice on sustainable approaches to weight loss.<br><br>Week 4: Healthy eating for families (Mums attend) - Provides advice on appropriate portion sizes for the whole family, discusses strategies for implementing the trust paradigm to encourage their children to eat healthily at home.<br><br>Week 5: The unique and powerful influence of fathers - Explains to dads why they have such a powerful influence over their kids, the importance of being a good role model and outlines the most effective parenting style.<br><br>Week 6: Raising active kids in an inactive world - Explains the growing issues of childhood obesity and why physical activity is so important for kids, highlights key strategies for dads to be physical activity leaders.<br><br>Week 7: 'Switching on' your child's mind by 'Switching off' - Highlights the physical and mental health issues created by excessive screen time and provides strategies for 'switching off'.<br><br>Week 8: 'Healthy' fathering in a busy world - Encourages discussion of barriers and solutions for achieving SMART goals, highlights opportunities to create family traditions and maximize the time dads can spend with their kids.<br><br>Children's sessions:<br><br>Week 1: Rough and tumble play - children learn about their mission to 'get dad fit and healthy' and are taught about rough and tumble activities.<br><br>Week 2: Turning Dad into a healthy eater - Through fun activities, kids learn about 'sometimes' foods and 'anytime' foods and how they can encourage dad to eat more healthily.<br><br>Week 3: Helping dad make healthy choices - children came up with suggestions for their Dad's packed lunch and were introduced to goal setting.<br><br>Week 4: The HDHK rainbow plate - Through fun activities, children learn about different fruits and vegetables and are challenged to make their plates 'rainbows' with a variety of healthy fruits and vegetables.<br><br>Week 5: Fun times with Dad - children are given activities to help them think about games they can play with dad to spend quality time together.<br><br>Week 6: Let's get active - children learning about importance of being active and considering how to stay active.<br><br>Week 7: Helping Dad 'Switch off' - children think about activities they could enjoy with Dad instead of playing on the computer or watching TV.

For peer review only

For peer review only - http://bmjopen.bmj.com/site/about/guidelines.xhtml

	Week 8: Becoming Dad's personal trainer - children develop an activity board with games and exercises the family can complete at home.<br><br>Week 9: Helping dad stay on track<br>**45-90 minutes. Fathers and children joint physical activity session**<br><br>Each week the joint father and child physical activity session covered three components:<br><br>- Rough and Tumble Play – *hands on activities played by each participant with their child, such as 'sock wrestle' (trying to get each other's socks off), 'stand up Dad' (children would try to stop their Dad standing up)*<br>- Fundamental skill development (for children) – *Coached activities focusing on child skill development of FMS such as catching, throwing, jumping, running etc.*<br>- Fitness activity (for fathers) – *Fitness activity focusing on fathers' fitness with support from children (e.g. Child helps count Dad's press ups, or sits on Dads back to make it harder or family running games racing each other).*
5. Who provided?	Three facilitators were required to deliver the HDHK programme. One facilitator to deliver the father-only education sessions and two facilitators to deliver to child-only education and joint father and child activity sessions.<br><br>Staff were recruited from four organisations to deliver the programme across the different courses. A face to face training session, a course manual and all materials required for delivery were provided.<br><br>The fathers-only education session facilitators were delivered by:<br><br>- Local Authority Healthy lifestyle Team (health trainer - female) – course 1<br>- Leisure centre staff employees (health and lifestyles manager and gym manager – both male) – course 2&3<br>- Sports coach of a national coaching organisation (female) – course 4<br><br>For successful delivery father-education facilitators were required to have presentation and group facilitation skills. The programme was designed for this section to be delivered by male facilitators, however due to staff availability this was not always possible.<br><br>The child-only education sessions and joint child and family activity sessions were delivered by:<br><br>- Self-employed fitness instructor - course 1<br>- Sports coaches – courses 2, 3 & 4.<br><br>Whilst not essential for delivery, sports coaching skills supported successful delivery to the physical activity elements of the programme. For child protection and ease of facilitation it was recommended that two facilitators ran the child-only education sessions. The second facilitator does not need to lead the session

For peer review only - http://bmjopen.bmj.com/site/about/guidelines.xhtml

	and this role could be undertaken by research staff or work experience students.
6: How	HDHK-UK is a group based programme, optimum group size 8-15 fathers
7. Where?	The programme is delivered in community venues such as community, youth or leisure centres and schools. The venue must have classroom (or equivalent) space for an educational session with PowerPoint projection facilities and a sports hall (or equivalent) for the physical activity session.
8. When and how often?	The programme is 9-weeks long with one 90-minute session delivered each week. Sessions need to be delivered at a time fathers and their children (aged 4-11 years) can attend. Of the four courses delivered, two were on a weekday evening, two were on a weekend. The weekend sessions were more popular.
9. Tailoring	Each week throughout the education session fathers were encouraged to reflect on the session content and apply the key messages in context to their own personal circumstances. Similarly, participants were asked to tailor their weekly goals and set home activities to meet their own and their family needs.
Modifications	Details of the programme adaptation from the original Australian programme are presented earlier in this chapter. Throughout the delivery of the adapted programme small modifications were made between courses, mainly where delivery of the father-only education session took longer than the allocated 30 minutes the material was shortened to ensure there was sufficient time for the physical activity session.
How well the programme was delivered -planned	The university research team completed observations of intervention delivery, completing a checklist to report on items of fidelity, including session timings, the delivery of key messages, facilitator skills and enthusiasm and participants responsiveness.
How well the programme was delivered - actual	Researcher observations of programme delivery showed that it was delivered with good fidelity. The main challenge was keeping the father-only education session to the allotted 30-minutes. This was improved throughout the courses. The fidelity of intervention delivery is described in Chapter 6.

For peer review only

For peer review only - http://bmjopen.bmj.com/site/about/guidelines.xhtml

Web-table 2: Baseline characteristics for the completers and non-completers at 6 months

	**Non-completers at 6 months** (N=16)	**Completers at 6 months** (N=27)	**Overall** (N=43)
**Minimisation Variables**			
Ethnicity			
White British	10 (62.5%)	7 (25.9%)	17 (39.5%)
Non White British	6 (37.5%)	20 (74.1%)	26 (60.5%)
**Demographics**			
Age (years)			
Mean (SD), N	38.7 (7.7), 16	40.7 (5.5), 27	40.0 (6.4), 43
Minimum-Maximum	23.6-56.0	31.6-52.8	23.6-56.0
Weight (kg)			
Mean (SD), N	93.4 (16.0), 16	89.2 (14.9), 27	90.8 (15.3), 43
Minimum-Maximum	70.9-136.9	64.0-142.5	64.0-142.5
BMI (kg/m²)			
Mean (SD), N	30.9 (6.1), 16	29.7 (4.4), 27	30.2 (5.1), 43
Minimum-Maximum	25.1-50.9	23.8-45.5	23.8-50.9
Index of Multiple Deprivation quintile, n (%)			
1 (least deprived)	1 (6.3%)	0 (0.0%)	1 (2.3%)

For peer review only - http://bmjopen.bmj.com/site/about/guidelines.xhtml

For peer review only

	**Non-completers at 6 months** (N=16)	**Completers at 6 months** (N=27)	**Overall** (N=43)
2	2 (12.5%)	0 (0.0%)	2 (4.7%)
3	2 (12.5%)	4 (14.8%)	6 (14.0%)
4	2 (12.5%)	7 (25.9%)	9 (20.9%)
5 (most deprived)	9 (56.3%)	14 (51.9%)	23 (53.5%)
Missing	0 (0.0%)	2 (7.4%)	2 (4.7%)

Note: All figures presented are N (%) unless otherwise specified.

For peer review only

For peer review only - http://bmjopen.bmj.com/site/about/guidelines.xhtml

CONSORT 2010 checklist of information to include when reporting a randomised trial*

Section/Topic	Item No	Checklist item	Reported on page No
**Title and abstract**			
	1a	Identification as a randomised trial in the title	1
	1b	Structured summary of trial design, methods, results, and conclusions (for specific guidance see CONSORT for abstracts)	2
**Introduction**			
Background and objectives	2a	Scientific background and explanation of rationale	4
	2b	Specific objectives or hypotheses	4
**Methods**			
Trial design	3a	Description of trial design (such as parallel, factorial) including allocation ratio	4
	3b	Important changes to methods after trial commencement (such as eligibility criteria), with reasons	n/a
Participants	4a	Eligibility criteria for participants	4-5
	4b	Settings and locations where the data were collected	4
Interventions	5	The interventions for each group with sufficient details to allow replication, including how and when they were actually administered	5 & supplementary web table
Outcomes	6a	Completely defined pre-specified primary and secondary outcome measures, including how and when they were assessed	6 - 7
	6b	Any changes to trial outcomes after the trial commenced, with reasons	n/a
Sample size	7a	How sample size was determined	5
	7b	When applicable, explanation of any interim analyses and stopping guidelines	n/a
Randomisation:			
Sequence generation	8a	Method used to generate the random allocation sequence	5
	8b	Type of randomisation; details of any restriction (such as blocking and block size)	5
Allocation concealment mechanism	9	Mechanism used to implement the random allocation sequence (such as sequentially numbered containers), describing any steps taken to conceal the sequence until interventions were assigned	5
Implementation	10	Who generated the random allocation sequence, who enrolled participants, and who assigned participants to interventions	5

For peer review only

For peer review only - http://bmjopen.bmj.com/site/about/guidelines.xhtml

Blinding	11a	If done, who was blinded after assignment to interventions (for example, participants, care providers, those assessing outcomes) and how	n/a
	11b	If relevant, description of the similarity of interventions	5
Statistical methods	12a	Statistical methods used to compare groups for primary and secondary outcomes	7 – 8
	12b	Methods for additional analyses, such as subgroup analyses and adjusted analyses	7 – 8
**Results**			
Participant flow (a diagram is strongly recommended)	13a	For each group, the numbers of participants who were randomly assigned, received intended treatment, and were analysed for the primary outcome	8, 9, Figure 1
	13b	For each group, losses and exclusions after randomisation, together with reasons	Figure 1
Recruitment	14a	Dates defining the periods of recruitment and follow-up	5
	14b	Why the trial ended or was stopped	n/a
Baseline data	15	A table showing baseline demographic and clinical characteristics for each group	Table 1
Numbers analysed	16	For each group, number of participants (denominator) included in each analysis and whether the analysis was by original assigned groups	Tables 1 – 3
Outcomes and estimation	17a	For each primary and secondary outcome, results for each group, and the estimated effect size and its precision (such as 95% confidence interval)	Tables 1 – 3
	17b	For binary outcomes, presentation of both absolute and relative effect sizes is recommended	Tables 1 – 3
Ancillary analyses	18	Results of any other analyses performed, including subgroup analyses and adjusted analyses, distinguishing pre-specified from exploratory	n/a
Harms	19	All important harms or unintended effects in each group (for specific guidance see CONSORT for harms)	14
**Discussion**			
Limitations	20	Trial limitations, addressing sources of potential bias, imprecision, and, if relevant, multiplicity of analyses	20 - 21
Generalisability	21	Generalisability (external validity, applicability) of the trial findings	20 – 21
Interpretation	22	Interpretation consistent with results, balancing benefits and harms, and considering other relevant evidence	20-21
**Other information**			
Registration	23	Registration number and name of trial registry	23
Protocol	24	Where the full trial protocol can be accessed, if available	Submitted
Funding	25	Sources of funding and other support (such as supply of drugs), role of funders	22

*We strongly recommend reading this statement in conjunction with the CONSORT 2010 Explanation and Elaboration for important clarifications on all the items. If relevant, we also recommend reading CONSORT extensions for cluster randomised trials, non-inferiority and equivalence trials, non-pharmacological treatments, herbal interventions, and pragmatic trials. Additional extensions are forthcoming: for those and for up to date references relevant to this checklist, see www.consort-statement.org.

For peer review only - http://bmjopen.bmj.com/site/about/guidelines.xhtml

BMJ Open

A weight management programme for fathers: Healthy Dads, Healthy Kids UK – a feasibility RCT

Journal:	*BMJ Open*
Manuscript ID	bmjopen-2019-033534.R1
Article Type:	Original research
Date Submitted by the Author:	03-Oct-2019
Complete List of Authors:	Griffin, Tania; University of Bath, Department for Health<br>Sun, Yongzhong; University of Birmingham, Birmingham Clinical Trials Unit (BCTU)<br>Sidhu, Manbinder S.; University of Birmingham, Health Services Management Centre<br>Adab, Peymane; University of Birmingham, Institute of Applied Health Research<br>Burgess, Adrienne; Fatherhood Institute<br>Collins, Clare; University of Newcastle, NSW, AU, School of Health Sciences<br>Daley, Amanda; Loughborough University, School of Sport, Exercise and Health Sciences<br>Entwistle, Andrew; Patient/Carer Reviewer,<br>Frew, Emma; University of Birmingham, Institute of Applied Health Research<br>Hardy, Pollyanna ; University of Birmingham, Birmingham Clinical Trials Unit<br>Hurley, Kiya; University of Birmingham, Cancer Research UK Clinical Trials Unit<br>Jones, Laura; University of Birmingham, Institute of Applied Health Research<br>McGee, Eleanor ; Birmingham Community Healthcare NHS Trust<br>Pallan, Miranda; University of Birmingham, Institute of Applied Health Research<br>Young, Myles; University of Newcastle, NSW, AU, School of Education<br>Morgan, Philip; University of Newcastle, NSW, AU, School of Education<br>Jolly, Kate; University of Birmingham, Institute of Applied Health Research
**Primary Subject Heading**:	Public health
Secondary Subject Heading:	Public health
Keywords:	Feasibility studies, Fathers, Weight loss, Healthy lifestyle

SCHOLARONE™
Manuscripts

A weight management programme for fathers: Healthy Dads, Healthy Kids UK – a feasibility RCT

Authors:

Tania Griffin[1], Yongzhong Sun[2], Manbinder Sidhu[3], Peymane Adab[4], Adrienne Burgess[5], Clare Collins[6], Amanda Daley[7], Andrew Entwistle[8], Emma Frew[4], Pollyanna Hardy[2], Kiya Hurley[9], Laura Jones[4], Eleanor McGee[10], Miranda Pallan[4], Myles Young[11], Philip J Morgan[11], Kate Jolly[4],

Corresponding author: Kate Jolly (c.b.jolly@bham.ac.uk)

1. Department for Health, University of Bath, Bath, UK
2. Birmingham Clinical Trials Unit, Institute of Applied Health Research, University of Birmingham, Birmingham, UK
3. Health Services Management Centre, University of Birmingham, Birmingham, UK
4. Institute of Applied Health Research, University of Birmingham, Birmingham, UK
5. The Fatherhood Institute, Marlborough, Wiltshire, UK
6. School of Health Sciences, Faculty of Health and Medicine, and Priority Research Centre for Physical Activity and Nutrition, University of Newcastle, New South Wales, Australia
7. School of Sport, Exercise and Health Sciences, Loughborough University, Loughborough, UK
8. Sandiacre, Sandy Lane, Leamington Spa, CV32 6QS
9. Cancer Research UK Clinical Trials Unit, Institute of Cancer and Genomic Sciences, University of Birmingham, Birmingham, UK
10. Birmingham B15 2TT Birmingham Community Health Care NHS Foundation Trust, Birmingham B7 4BN, UK
11. School of Education, and Priority Research Centre for Physical Activity and Nutrition, University of Newcastle, New South Wales, Australia

Keywords: Feasibility Studies, Fathers, Weight loss, Healthy Lifestyle

Word Count: 4256

For peer review only - http://bmjopen.bmj.com/site/about/guidelines.xhtml

ABSTRACT

Objectives: To assess (i) the feasibility of delivering a culturally adapted weight management programme; Healthy Dads, Healthy Kids United Kingdom (HDHK-UK) for fathers with overweight or obesity and their primary school aged children, and (ii) the feasibility of conducting a definitive randomised controlled trial.

Design: A two-arm, randomised feasibility trial with a mixed-methods process evaluation.

Setting: Socio-economically disadvantaged, ethnically diverse localities in West Midlands, UK.

Participants: Fathers with overweight or obesity and their 4-11-year old children.

Intervention: Participants were randomised in a 1:2 ratio to control, (family voucher for a leisure-centre) or intervention comprising nine weekly healthy lifestyle group sessions.

Outcomes: Feasibility of the intervention and RCT were assessed according to pre-specified progression criteria: study recruitment, consent and follow-up, ability to deliver intervention, intervention fidelity, adherence and acceptability, weight loss, using questionnaires and measurements at baseline, 3 and 6-months, and through qualitative interviews.

Results: The study recruited 43 men, 48% of the target sample size; mean BMI 30.2kg/m^2 (SD 5.1), 61% were from a minority ethnic group and 54% from communities in the most disadvantaged quintile for socio-economic deprivation. Recruitment was challenging. Retention at 3 and 6-month follow-up was 63%. Identifying delivery sites and appropriately skilled and trained programme facilitators proved difficult. Four programmes were delivered in leisure-centres and community venues. Of the 29 intervention participants, 20 (69%) attended the intervention at least once, of whom 75% attended ≥5 sessions. Sessions were delivered with high fidelity. Participants rated sessions as 'good/very good' and reported lifestyle behavioural change. Weight loss at 6 months in the intervention group (n=17) was 2.9kg (95%CI -5.1 to -0.6).

Conclusions: The intervention was well-received, but there were significant challenges in recruitment, programme delivery and follow-up. The HDHK-UK study was not considered feasible for progression to a full RCT based on pre-specified stop-go criteria.

Trial registration: ISRCTN16724454

For peer review only - http://bmjopen.bmj.com/site/about/guidelines.xhtml

Strengths and limitations of this study

- This unique study provides new evidence into the feasibility of delivery of a weight management programme targeting fathers from socio-economically deprived, ethnically diverse community settings, and its acceptability in this target population, a key strength given the relative paucity of research in this area.
- Recruitment methods were wide ranging, raising awareness of the study in a number of community settings, however despite extensive efforts recruitment to the study was a key challenge.
- The intention was to recruit male facilitators to deliver the intervention sessions, however it was difficult to identify suitable facilitators with the necessary skill set, therefore a range of male and female facilitators were recruited.
- Identifying a convenient time to run the intervention course to suit fathers, venue and facilitator availability proved challenging.

For peer review only - http://bmjopen.bmj.com/site/about/guidelines.xhtml

INTRODUCTION

Overweight and obesity remain a public health challenge and priority.[1] At an individual level excess weight results in fewer disease-free years and lowered life expectancy,[2, 3] and at a population level obesity prevalence is associated with major economic burden.[4]

In England, overweight and obesity levels are higher in men compared to women with socioeconomic inequalities evident.[5] Men from the lowest income quintile have a greater mean waist circumference than men from the highest income quintile.[6] South Asian men have higher body fat percentage than White Europeans, at the same body mass index (BMI).[7]

The transition to fatherhood has been identified as a critical period during which men are more likely to adopt obesogenic behaviours and are more susceptible to weight gain.[8, 9] While there have been several successful men-only weight management programmes,[10-14] attendance for such activities remains higher amongst women.[13] Fathers are also under-represented in interventions targeting family health behaviours.[15] This emphasises the need to identify programmes which fathers are keen to engage with.

Healthy Dads Healthy Kids (HDHK) is a weight management programme for fathers of primary school aged children that also addresses the healthy eating and physical activity behaviours of their children. The programme, developed and delivered in Australia, was shown to be successful at achieving weight loss in fathers.[16, 17] In the current study, the HDHK programme was adapted with the intention of making it culturally acceptable to a multi-ethnic UK population.[18] The aim of this study was to assess the feasibility of delivering the adapted HDHK programme and the feasibility to conduct a future RCT in a socioeconomically deprived ethnically diverse UK setting.

METHODS

Study design and participants

This study was a two-arm, randomised feasibility trial with a mixed-methods process evaluation. It was conducted in two urban local authority areas of the West Midlands, UK, (site 1 and site 2) selected for their population profile and interest in supporting the programme. In 2017, both areas were ranked in the most deprived 20% of areas in the UK, with high ethnic diversity.[19]

The study aimed to recruit men who were father/step-father/father figures (herein referred to as ‘fathers’) of primary school children (aged 4-11 years), aged 18-65 years with a BMI

For peer review only - http://bmjopen.bmj.com/site/about/guidelines.xhtml

≥25kg/m^2 (≥23kg/m^2 for minority ethnic groups)[7] and/or a waist circumference of ≥94cm (37 inches) who were willing to lose weight. Fathers did not have to be co-resident with their child(ren). Men were not eligible if they had a history of angina or other cardiovascular disease; orthopaedic or joint problems that would be a barrier to vigorous physical activity; weight loss of 3kg/7lbs in the previous three months; current diabetes and were not confident in managing their condition during exercise; were unable to speak and/or understand English; involved in ongoing custody or access disputes and/or any contexts with a risk of domestic violence.

Sample size

As a feasibility study, no formal sample size calculation was performed. We aimed to recruit 90 men and their children to estimate the recruitment, follow-up and questionnaire completion rates to within +/- 10% with 95% confidence, based on a worst case estimate of 50%.

Recruitment and randomisation

Fathers were recruited (September 2017-January 2018) by the research team who had extensive experience of participant recruitment in a community setting. A range of methods were used over the recruitment period including flyer distribution and promotion stands at leisure, community and shopping centres, places of worship and large workplace organisations. Recruitment via schools conducted through presentations at school assemblies and teacher meetings, stands at parent evenings, flyer distribution and talking to parents at school pick up time. The study was promoted on social media (Twitter and Facebook).

Written informed consent was obtained from fathers and assent from children aged 8 years and over. Once baseline data were collected, participants were randomised (1:2 allocation ratio) to the control group (voucher for a single family visit to a leisure centre) or intervention group (adapted HDHK-UK programme). Randomisation was stratified by father's ethnicity (white British or Irish / other ethnic group) and conducted using an automated online form developed by University of Birmingham Clinical Trials Unit.

Intervention

The HDHK-UK intervention comprised weekly 90-minute sessions over nine consecutive weeks; four courses were delivered. Fathers and children attended all sessions which followed the same structure: 15 minutes' discussion and review of the weekly activities followed by 30 minutes where children and fathers took part in an education session separately. The groups were facilitated by local, experienced and trained staff to ensure the sessions were interactive and discussion was encouraged. Facilitators were selected based on their experience of delivering group programmes and delivering health advice, they included health trainers (who

For peer review only - http://bmjopen.bmj.com/site/about/guidelines.xhtml

provide community support for health related behaviour changes) and sports coaches. They completed HDHK delivery training with either the Fatherhood Institute or the research team. The training included practicing delivery of parts of the intervention. Fathers' sessions covered a range of lifestyle behaviours around the importance of physical activity, nutrition and parenting. Children were taught about healthy eating, physical activity and how to be a supportive family member by encouraging and modelling healthy lifestyle behaviours at home. The final 45 minutes of the session were spent doing physical activity within family groups. These practical sessions had three elements: 'rough and tumble' play; teaching children fundamental movement skills (catching, throwing, kicking etc.); and aerobic fitness. The intervention is summarised in TIdIER checklist (web-table 1). The HDHK intervention draws on concepts from Family Systems Theory[20, 21] and Social Cognitive Theory;[22] the theoretical constructs are reported for the original programme.[17] Adaptations to the Australian resources were made by the research team (KJ and MS) and one of the study partners (The Fatherhood Institute) in conjunction with the wider research team. The adaptations were informed by qualitative research with fathers and mothers from similar socio-economic backgrounds and residing in the same geographical region.[23] Adaptations focussed on reducing the number of PowerPoint slides, simplifying and anglicising wording and updating the guidance and statistics to align to UK public health recommendations. References to foods, activities and images were updated to reflect a multi-cultural UK population. Fathers and their children attended every session and mothers were invited to one session when family food was discussed.

Data collection and outcomes

Data were collected from participants by trained researchers at baseline, and three- and six-months later in their home (or convenient location). Sociodemographic characteristics were collected by questionnaire. Based on postal code of residence, the Index of Multiple Deprivation (IMD) was used to determine socioeconomic status (SES).[24]

Primary outcomes: feasibility measures

The outcomes relating to feasibility of delivery of HDHK-UK were: ability to recruit and retain facilitators, deliver sessions at a time and location convenient for participants, fidelity of delivery, and acceptability to participants.

The outcomes relating to the feasibility of conducting a future definitive RCT were: recruitment rate, willingness to be randomised, follow-up rates, level of completion of follow-up questionnaires, and father's weight change in the intervention group.

For peer review only - http://bmjopen.bmj.com/site/about/guidelines.xhtml

Process evaluation

Process measures were collected by the research team to determine the feasibility of intervention delivery and study processes, fidelity of intervention delivery and participant acceptability. Sixteen session observations (minimum of two per intervention course) were completed to assess content delivery and participant engagement. Participants and facilitators were also asked to complete feedback forms after each session, evaluating the session content and delivery. Qualitative interviews were conducted with 12 participants post-intervention. One participant was interviewed at both 3 and 6-months. The average interview duration was 16 minutes and interviews were conducted either face to face (n=4) or by telephone (n=9). All facilitators (n=7) were interviewed, five interviews were conducted individually, one with two facilitators together.

Secondary outcome measures

Secondary outcome measures for fathers were outcomes that would be collected in the main trial (if progressed to). These comprised: percentage losing ≥5% body mass, change in waist circumference and percent body fat, self-reported physical activity (using the IPAQ-short[25]), objectively measured physical activity (by a wrist worn GeneActiv© (Activinsights, Cambs, United Kingdom), triaxial accelerometer worn for 7-days on the father's non-dominant wrist), self-reported dietary intake (using food frequency items (FFQ) items), father-child relationship outcomes (using the Parent-Child Relationships Questionnaire),[26] parenting for physical activity (using the Parenting Strategies for Eating and Activity Scale[27] and the Physical Activity Modelling subscale of the Activity Support Scale for Multiple Groups (ACTS-MG)[28]).

Secondary outcome measures collected from the children comprised BMI z-score change (calculated using the LMS method[29] and UK reference data[30]) percent body fat, overweight or obese, objectively measured physical activity (GeneActiv© worn by the eldest child only), parent-reported dietary intake for the eldest child (using the Family Nutrition and Physical Activity questionnaire[31]) and behaviour and emotional well-being (using the Strength and Difficulties questionnaire).[32]

To assess the feasibility of collecting outcome data for a future health economic analysis we collected the EQ-5D-5L[33] and ICECAP-A[34] from fathers and the CHU-9D[35-37] from the children.

Progression criteria

Progression criteria, agreed by the funding panel and Study Steering Committee, were pre-defined to help evaluate whether the feasibility trial should be recommended to progress to a fully powered RCT. These are detailed in the results section.

For peer review only - http://bmjopen.bmj.com/site/about/guidelines.xhtml

Data analysis

For the quantitative analyses, the aim was to assess the progression criteria and feasibility of delivery of a main trial. All analyses were by intention-to-treat and undertaken in Stata V.12. Feasibility outcomes are presented overall and by group with counts, percentages and 95% confidence intervals. Weight change in fathers at 3 and 6-months follow-up are summarised using means, standard deviations and within group 95% confidence intervals. Baseline characteristics are summarised by group and overall and the proposed secondary outcomes of a definitive trial are summarised by group only. Categorical data are presented using counts and percentages, and continuous data are presented using means and standard deviations or median and interquartile range where appropriate. Since the study was not powered to detect treatment effects on clinical outcomes, p-values and 95% confidence intervals are not reported.

Audio recordings of qualitative interviews were transcribed verbatim, anonymised and analysed using the framework approach.[38]

Public involvement

A public and patient participation group of eight fathers and two mothers from one of the research sites w ere involved throughout all stages of the study by contributing to decisions about outcome measures, commenting on intervention materials (in particular the need for their simplification), and participant facing documents (downplaying the focus on weight management and focussing on the opportunity for father-child interaction in recruitment materials), advising on recruitment strategies and dissemination of the findings.

RESULTS

Feasibility of conducting a definitive RCT:

Despite multiple recruitment methods, only 92 men expressed interest in the study, of whom 43 were recruited and randomised (intervention group n=29) (Figure 1); 62 children were recruited. No participants declined randomisation after an assessment visit, nor was randomisation given as a reason for declining after expressing an interest in the study.

Four HDHK-UK courses were delivered, one at site 1 (in a youth centre) and three at site 2 (two in a leisure centre and one in a community centre).

Baseline characteristics of participants

For peer review only

The fathers' mean BMI at baseline was 30.2kg/m^2 (SD 5.1) and their mean age was 40.0 years (range 23.8-56.0). The mean age of their participating children (n=62) was 7.7 years (range 4.0–11.7) of whom 20 (32.8%) were overweight or obese. Overall, 60.5% of participants were from minority ethnic groups and 74.4% lived in the two most deprived quintiles of the IMD. Details of the baseline characteristics are presented in Table 1.

For peer review only

For peer review only - http://bmjopen.bmj.com/site/about/guidelines.xhtml

Table 1: Baseline characteristics for fathers and children by treatment arm

	HDHK-UK programme (N=29)	Minimum Intervention (N=14)	Overall (N=43)
**Fathers**			
Age (years)			
Mean (SD), N	39.4 (6.3), 29	41.1 (6.6), 14	40.0 (6.4), 43
Ethnicity			
White British	12 (41.4%)	5 (35.7%)	17 (39.5%)
Non White British	17 (58.6%)	9 (64.3%)	26 (60.5%)
Highest level of qualification			
GCSE, CSE, O level or equivalent	7 (24.1%)	6 (42.9%)	13 (30.2%)
A-level/AS level or equivalent	3 (10.3%)	2 (14.3%)	5 (11.6%)
Degree level or higher	15 (51.7%)	6 (42.9%)	21 (48.8%)
Other	2 (6.9%)	0 (0.0%)	2 (4.7%)
Missing	2 (6.9%)	0 (0.0%)	2 (4.7%)
Legal marital or civil partnership status			
Married or in a registered civil partnership	25 (86.2%)	13 (92.9%)	38 (88.4%)
Divorced or formerly in a civil partnership which is now legally dissolved	1 (3.5%)	0 (0.0%)	1 (2.3%)
Never married and never registered in a civil partnership	1 (3.5%)	1 (7.1%)	2 (4.7%)
Missing	2 (6.9%)	0 (0.0%)	2 (4.7%)
Main spoken language			
English	23 (79.3%)	14 (100.0%)	37 (86.1%)
Urdu	1 (3.5%)	0 (0.0%)	1 (2.3%)
Punjabi	1 (3.5%)	0 (0.0%)	1 (2.3%)
Spanish	1 (3.5%)	0 (0.0%)	1 (2.3%)
Turkish	1 (3.5%)	0 (0.0%)	1 (2.3%)
Missing	2 (6.9%)	0 (0.0%)	2 (4.7%)
Index of Multiple Deprivation quintile, n (%)			
1 (least deprived)	1 (3.5%)	0 (0.0%)	1 (2.3%)
2	1 (3.5%)	1 (7.1%)	2 (4.7%)
3	5 (17.2%)	1 (7.1%)	6 (14.0%)
4	5 (17.2%)	4 (28.6%)	9 (20.9%)
5 (most deprived)	17 (58.6%)	6 (42.9%)	23 (53.5%)
Missing	0 (0.0%)	2 (14.3%)	2 (4.7%)
BMI (kg/m²)			
Mean (SD), N	30.1 (4.8), 29	30.2 (5.8), 14	30.2 (5.1), 43
Weight at baseline (kg)			
Mean (SD), N	90.1 (13.2), 29	92.2 (19.3), 14	

For peer review only - http://bmjopen.bmj.com/site/about/guidelines.xhtml

	HDHK-UK programme (N=29)	Minimum Intervention (N=14)	Overall (N=43)
Percentage body fat (%)			
Mean (SD), N	28.3 (7.6), 28	29.8 (9.0), 14	28.8 (8.0), 42
Waist circumference (cm)			
Mean (SD), N	101.8 (8.5), 19	103.1 (15.0), 14	102.3 (11.5), 33
International physical activity questionnaire[25] (IPAQ-short)			
Low activity	8 (27.6%)	6 (42.9%)	14 (32.6%)
Moderate activity	10 (34.5 %)	4 (28.6%)	14 (32.6%)
Vigorous activity	10 (34.5%)	3 (21.4%)	13 (30.2%)
Missing	1 (3.5%)	1 (7.1%)	2 (4.7%)
Total activity (minutes) from GENEactiv measurement			
Median [IQR]	207.3 [175.8, 270.1]	213.6 [157.4, 248.9]	210.4 [167.7, 264.6]
Missing	4	1	5
Moderate/vigorous activity (minutes) from GENEactiv measurement			
Median [IQR]	110.8 [88.4-141.3]	87.0 [75.9-148.4]	109.7 [83.8-148.4]
Missing	4	1	5
EQ-5D-5L Index score[33] (adult Quality of Life)			
Mean (SD)	0.91 (0.16)	0.95 (0.07)	0.93 (0.13)
Missing	2	0	2
ICECAP-A total capability score[34] (adult wellbeing and Quality of Life)			
Mean (SD)	0.86 (0.15)	0.95 (0.06)	0.89 (0.13)
Missing	1	0	1
**Children**			
	**(N=42)**	**(N=19)**	**(N=61)**
Age (years)			
Mean (SD)	7.7 (2.0)	7.8 (2.2)	7.7 (2.1)
Minimum-Maximum	4.2-11.5	4.0-11.7	4.0-11.7
Sex			
Female	16 (38.1%)	6 (31.6%)	22 (36.1%)
Male	26 (61.9%)	13 (68.4%)	39 (63.9%)
BMI (kg/m²)			
Underweight/healthy	20 (47.6%)	13 (68.4%)	33 (54.1%)
Overweight/obese	16 (38.1%)	4 (21.1%)	20 (32.8%)
Missing	6 (14.3%)	2 (10.5%)	8 (13.1%)
Percentage body fat (%)			
Mean (SD)	24.0 (5.8)	22.9 (4.9)	23.6 (5.5)
Minimum-Maximum	10.3-36.7	16.9-37.3	10.3-37.3

For peer review only - http://bmjopen.bmj.com/site/about/guidelines.xhtml

	**HDHK-UK programme** (N=29)	**Minimum Intervention** (N=14)	**Overall** (N=43)
Missing	6	3	9

Note: All figures presented are N (%) unless otherwise specified.
BMI: body mass index; IQR: interquartile range; SD: standard deviation

Follow-up rates

The follow-up rate at three- and six-months post intervention was 62.8% (n=27); 58.6% (n=17) in the intervention group and 71.4% (n=10) in the control group (figure 1). Participants lost to follow-up at 6-months were more likely to be white British (supplementary web-table 2).

Level of completion of follow-up questionnaires

Researchers experienced challenges in arranging appointments for data collection. Despite repeated reminders, researchers often arrived at a family's house to find they were no longer available. Work and after-school activities made finding times for recruitment visits difficult and impacted recruitment numbers. For fathers and their children who completed data collection appointments, the processes worked well. In the main, the completeness of the data was acceptable with the exception of high levels of missing data for the IPAQ questionnaire and fathers' waist circumference, which was frequently refused (23% missing at baseline).

Fathers' weight change in the intervention group

Table 2 summarises fathers' weight change from baseline. The intervention group had a 2.9kg (95% CI 0.6 to 5.1) reduction in weight at 6-months follow-up.

For peer review only - http://bmjopen.bmj.com/site/about/guidelines.xhtml

Table 2 Weight change and secondary outcome measures for fathers by treatment arm

	3-months		6-months	
	**HDHK UK programme** (N=29)	**Minimum Intervention** (N=14)	**HDHK UK programme** (N=29)	**Minimum Intervention** (N=14)
**Weight change from baseline (kgs)**				
Mean (SD), N [95% CI]	-1.8 (2.5), 17 [-3.1 to -0.5]	-1.2 (3.3), 9 [-3.7 to 1.3]	-2.9 (4.1), 15 [-5.1 to -0.6]	-2.0 (3.6), 10 [-4.6 to 0.6]
**Change from baseline in waist circumference (cm)**				
Mean [SD], N	-10.8 (18.0), 9	3.4 (9.3), 6	-5.2 (5.0), 6	-2.8 (6.4), 5
**Change from baseline in % body fat**				
Mean [SD], N	-1.5 (3.1), 16	-0.3 (2.6), 9	-2.2 (3.2), 14	-2.3 (3.9), 9
**Physical activity measured by a GENEactive accelerometer**				
Median for total activity (minutes) [IQR], N	208.8 [185.6, 287.0], 17	168.3 [147.8, 194.0], 9	239.6 [194.3, 287.0], 11	146.2 [125.0, 230.6], 9
Median for moderate/vigorous activity [IQR], N	113.3 [99.3, 151.3], 17	84.2 [69.4, 106.2], 9	125.6 [101.3, 163.1], 11	68.2 [59.1, 86.5], 9
**EQ-5D-5L**[33] (adult health related quality of Life)				
Mean [SD], N	0.87 (0.19), 17	0.94 (0.10), 9	0.92 (0.14), 14	0.94 (0.13), 9
**ICECAP-A**[34] (adult capability and wellbeing)				
Mean [SD], N	0.92 (0.09), 17	0.95 (0.05), 9	0.89 (0.13), 14	0.92 (0.07), 9
**Lost ≥5% body mass**				
Yes	2 (6.9%)	1 (7.1%)	9 (31.0%)	1 (7.1%)
No	15 (51.7%)	8 (57.1%)	6 (20.7%)	9 (64.3%)
Missing	12 (41.4%)	5 (35.7%)	14 (48.3%)	4 (28.6%)
**Parenting for physical activity**				

For peer review only

Activity Support scale for Multiple Groups (ACTS-MG)[28]				
Mean (SD), N	15.6 (3.5), 17	13.0 (2.3), 9	16.4 (2.2), 14	13.7 (1.5), 9
Parenting strategies for Eating and Activity Scale[27] (Mean (SD), N)				
Limit setting	8.4 (1.9), 17	8.8 (1.4), 9	7.5 (2.1), 13	9.1 (1.2), 9
Control	2.7 (1.6), 17	2.1 (1.3), 9	3.4 (1.3), 13	2.6 (1.7), 9
Monitoring	7.3 (1.5), 17	7.4 (1.1), 9	7.4 (1.5), 14	7.7 (0.7), 9
Disciplining	5.2 (2.3), 17	4.7 (2.4), 9	5.2 (2.7), 14	6.3 (2.2), 9
Co-physical activity	3.5 (0.9), 17	2.7 (0.7), 9	3.8 (1.3), 14	3.2 (0.8), 9
**Father-child relationship[26]** (Mean (SD), N)				
[a]Disciplinary warmth	22.9 (4.0), 16	23.0 (1.6), 9	23.8 (3.6), 14	23.3 (3.0), 9
[b]Personal relationships	31.4 (5.0), 16	28.9 (2.6), 9	30.6 (4.8), 14	30.0 (2.9), 9

BMI: body mass index; EQ-5D-5L: EuroQol; ICECAP-A: ICEpop CAPability measure for Adults; IQR: interquartile range; SD: standard deviation;

[a]Disciplinary warmth= praise + shared decision making + rationale; [b]Personal relationships (pro-social + intimacy + nurturance + companionship)

For peer review only

Secondary outcomes

Table 2 summarises other secondary outcomes for fathers and Table 3 for the children. The intervention group fathers had favourable reduction in waist circumference; 31% (n=9) achieved a 5% reduction in body mass at 6-months. There were no serious adverse events requiring hospital admission, or adverse events requiring medical attention during the intervention.

For peer review only

For peer review only - http://bmjopen.bmj.com/site/about/guidelines.xhtml

Table 3: Secondary outcome measures for eldest child by treatment arm

	3-months		6-months	
	**HDHK UK programme** (N=29)	**Minimum Intervention** (N=14)	**HDHK UK programme** (N=29)	**Minimum Intervention** (N=14)
**Change from baseline in BMI z-score**				
Mean [SD], N	-0.131 (0.272), 14	0.016 (0.346), 8	-0.016 (0.299), 12	0.039 (0.449) ,10
**Change from baseline in % body fat**				
Mean [SD], N	-0.58 (1.17), 13	0.04 (2.84), 8	-0.80 (1.48), 11	-0.88 (3.07), 8
**Categorised as overweight or obese (%)**				
Underweight/healthy	8 (27.6%)	6 (42.9%)	7 (24.1%)	6 (42.9%)
Overweight/obese	7 (24.1%)	3 (21.4%)	6 (20.7%)	4 (28.6%)
Missing	14 (48.3%)	5 (35.7%)	16 (55.2%)	4 (28.6%)
**Physical activity measured by a GENEactive accelerometer**				
Median for total activity (minutes) [IQR], N	342.3 [262.7, 427.7], 17	277.0 [272.5, 314.3], 9	347.3 [321.6, 384.0], 10	312.8 [245.4, 456.8], 8
Median for moderate/vigorous activity [IQR], N	73.5 [34.7, 99.3], 17	57.0 [26.8, 73.0], 9	73.2 [49.0, 105.7], 10	56.0 [32.4, 110.6], 8
**Family nutrition & physical activity questionnaire[31]**				
Mean [SD], N	62.3 (7.7), 16	61.1 (5.2), 9	61.2 (6.5), 9	62.1 (3.0), 8
**CHU-9D[35-37]** (child utility measure)				
Mean [SD], N	0.89 (0.09), 15	0.93 (0.04), 9	0.92 (0.09), 11	0.92 (0.11), 8
**[a]Strengths and difficulties questionnaires[32] (Total SDQ)**				
Mean [SD],N	-	-	7.4 (3.8), 12	11.2 (6.1), 6

[a]SDQ: Each 1 point increase in the total difficulties score corresponds with an *increase in the risk* of developing a mental health disorder.

BMI: body mass index; CHU-9D: Child Health Utility-9D

For peer review only - http://bmjopen.bmj.com/site/about/guidelines.xhtml

Feasibility and acceptability of programme delivery

Ability to recruit and retain facilitators

Site 1 provided health trainers employed by the local authority for the fathers' education component and an independent fitness trainer delivered the physical activity elements. At site 2, two courses were delivered in a leisure centre by their staff and one course was delivered in a community centre by sports coaches employed by a coaching organisation.

We experienced challenges in delivering training due to loss of potential facilitators as a result of rapid turnover of staff from the various delivery organisations. Three forms of training were delivered: a two-day HDHK training workshop delivered by the Fatherhood Institute, who had previously been trained by the Australian team; a half day of top-up training by the Fatherhood Institute; and more flexible training for facilitators recruited after these sessions, delivered by a member of the research team (TG) who had previous experience of coaching training.

We intended that two courses would be delivered at each site. However, due to organisational restructuring, the health trainers were only able to deliver one course.

Challenges to recruiting participants

There were significant challenges with recruitment. We obtained permission from 40 organisations to recruit on their premises (including 11 schools, 7 faith organisations, 6 sport/leisure centres), but it was a time consuming process. Communication with schools was slow and recruitment via children and 'mothers', was not a successful strategy. Fathers were hard to engage during recruitment activities and session timings were often not convenient for their family. Additionally, as a result of a change in sites after funding was awarded (due to a local site withdrawing funding for adult weight management services), the sites involved considerable researcher travel time.

Deliver sessions at a time and location convenient for participants

Aligning venue, facilitator and participant availability was a major challenge and resulted in considerable delays in commencing programme delivery. Evening sessions were difficult due to fathers' work commitments, and children's meal and bed times. Although weekends were reported to be convenient by participants, the availability of facilitators and community venues were more limited.

> *I wasn't finishing work until 5 o'clock and then I was having to fight my way through the traffic to get there. If it had been any later, it's harder for the kids then because they've*

For peer review only - http://bmjopen.bmj.com/site/about/guidelines.xhtml

got to get up for school the next week. ... and weekends wouldn't be any good because the leisure centre would be packed. (ID B-008)

Session timings were also the main reason given by participants for non-attendance at intervention sessions.

It started at half past five which is obviously rush hour time. Dads are leaving work ... In the end, what happened was it went from a group of six dads and their children to the last session being just three dads …. I think that wasn't due to their motivation but their work time commitments. (ID B-068)

Fidelity of delivery

The intervention was delivered with high fidelity. The style of delivery and the small group sizes meant group discussions were encouraged, allowing content to be tailored to the group's needs (facilitators were trained to manage group discussions as part of the HDHK delivery training); however facilitators reported finding it challenging to deliver all the session content in the allocated time. This was especially difficult if participants arrived to the sessions late and facilitators had to balance content delivery whilst also allowing for group discussion and interaction. Researchers sometimes observed some session content being skipped or a delay in the start of the subsequent practical session. Despite the challenges, the facilitator's ensured key content was delivered, which was verified in participant interviews where they spoke of the key messages the course had focused on.

Acceptability of adapted intervention to participants

Twenty participants (69%) attended an intervention course at least once; 15 attended (52%) at least five sessions. Fathers who attended the sessions and took part in a qualitative interview were positive about their experiences. Feedback sheets, observations and qualitative interviews with both participants and facilitators consistently showed high levels of acceptability towards the intervention programme.

It [HDHK-UK programme] was brilliant overall. I really enjoyed it. The kids enjoyed it. (ID B-068)

The group sessions were appreciated by participants:

I enjoyed the group-based elements … it allowed to people to bounce off, talk about - and also the blokes, a little bit of competitive edge, especially when the weight round was coming. So I think it benefited me and I preferred the group format rather than just individually between me and my children. (ID A-077)

A strong theme was the appreciation of time spent with their children "*I'm working during the week and it's just nice to have that dad-and-daughter time when just for a couple of hours it was just us and I think we've really benefited from that"* (ID A-072)'

Participants' spoke highly of the facilitators for the education sessions; *'Fantastic, very well presented, well engaging, knowledgeable.* (ID A-058)' and the physical activity sessions: '*they were excellent, really good lads…we were always busy, always sweating, it was all good'* (ID B-089). Similarly, the facilitators spoke positively of the programme.

A key challenge was delivery over the UK winter:

> *The weather had been against us a lot of the times, I think if we had done it now in the summer, I think it would have been a lot better for us (ID B-089)*
>
> *…although we've been out Saturdays and Sundays it's trying to fit it into darker nights when it's been cold and it's been wet or it's been snowing or whatever else, that's been difficult. (*ID A-072)

Progression criteria

Progression criteria for a full trial (Table 4) were met for intervention acceptability to participants who attended and fidelity of delivery, recruitment to the trial was low and attrition was high (37.2%, n=16). Mean weight loss in the intervention arm was marginally below the pre-specified ≥3kg in the participants who were followed-up.

For peer review only

Table 4 Progression criteria for a full trial

Progression criterion	Assessment
Intervention is acceptable to a majority of fathers and families from differing BAME and socio-economic backgrounds;	Achieved
Randomisation occurs and more than 80 per cent of those assessed accept randomisation;	Achieved
Recruitment of at least 68 out of the planned 90 fathers (75 per cent) within the 4-month time frame;	43 (48%)
Intervention implemented with fidelity in 75 per cent of observations (see 'process evaluation' section above);	Achieved
Attendance 70 per cent attending at least 5/9 of the planned sessions;	15 (52%)
More than 70 per cent follow up at three and six-months;	27 (62.8%)
Mean weight loss in the intervention arm of ≥3kg.	2.9kg (95% CI -5.1, -0.6)

DISCUSSION

The aims of the current study were to assess the feasibility of delivering a culturally adapted HDHK programme in a socioeconomically deprived UK setting, and the feasibility of conducting a definitive RCT. Overall, it was possible to recruit and train facilitators to deliver the intervention programme with high fidelity. The programme was rated highly by the attending participants and delivery teams and those who participated achieved weight loss comparable with other studies.[13, 16] However, recruitment of participants was difficult, and aligning participant, venue and facilitator availability to deliver the programme was a major challenge. As such, participant recruitment and attendance rates were low and the progression criteria for the study were not met.

Despite multiple strategies, encouraging fathers to engage with the study was challenging. Attempts to enhance recruitment through schools, an approach used in the Australian setting, proved more difficult than anticipated. Schools are often involved in multiple projects and programmes, which may have meant they felt they did not have added capacity for HDHK-UK. It was recognised during recruitment that there can be sensitivities around discussing weight. To try to offset this, the opportunity for fathers to spend time with their children was emphasised. Challenges in recruitment have also been recognised in a similar study with fathers[39] and as a common theme in a systematic review of weight loss trials in men.[13, 14] However, the findings and experiences from this current study differ from those of the

For peer review only - http://bmjopen.bmj.com/site/about/guidelines.xhtml

Australian HDHK RCT.[16, 17] Notably, the Australian study used a wait-list study design where all families were guaranteed to receive the programme at some point, which may have been more appealing for participants. An additional difference was a greater availability of sessions.

Nearly a third of participants allocated to the intervention group did not engage with the programme. This has been seen in other group based health related programmes[40, 41,42] but differs from the HDHK trial in Australia.[16, 17] The primary reason given for non-attendance or non-completion of the programme was that the session timings were not suitable, which was corroborated by the research team who found identifying a convenient time for programme delivery to be one of the most significant challenges of the study. Evening sessions had to align to fathers working patterns and children's school and bed-time routines, resulting in sessions often being set when traffic was at its heaviest. This was compounded by running the sessions in the winter with dark nights and poor weather. Weekend sessions often clashed with family activities and venue and facilitator availability were more limited. Poor weather is likely to have also impacted recruitment, especially at schools where parents were not keen to stop to talk to the study team in rainy or cold conditions. Delivery in summer and offering more choice of sessions might mitigate some of these challenges.

The facilitators reported it to be challenging to deliver the full content of the father's education session within the allocated 30 minutes, exacerbated by participants arriving late. The approach to sessions was to encourage interaction and discussion, which was appreciated by participants, and necessary if the group size was small. However, this at times challenged the fidelity of delivery and if the programme were to be rolled out we would recommend reducing session content further.

The youngest children participating in the current study had difficulty in engaging with written materials; four year olds were not eligible in the Australian studies.[16, 17, 42]

Strengths and Limitations

The study has a number of strengths. Whilst successful weight management trials in men have been reported[10, 12, 13, 16, 17] most participants in weight loss trials are women.[43, 44] In addition, very few specifically target ethnic minority population groups[43] or recruit from socio-economically disadvantaged populations, which is a unique contribution of this study.

This study also had limitations. First, the small number of participants recruited and attending the programme meant some adaptation for a smaller group size was required, but meant some of the group atmosphere was reduced. Those who attended enjoyed the programme, and whilst the remainder advised that session timing was their main reason for non-attendance,

For peer review only - http://bmjopen.bmj.com/site/about/guidelines.xhtml

the small sample size creates bias, especially for the qualitative interviews. Whilst every attempt was made to interview non-attendees and non-completers, engagement was low. We had also planned to interview participants with their partners and children present, but logistically this proved difficult to arrange; many of the children were too young to participate in an interview and most fathers opted for a phone interview as they found this more convenient.

Another limitation was the change in facilitation staff throughout the study, resulting in the need to run individualised training sessions after the original training delivered by the Fatherhood Institute. Whilst the additional training was delivered by an experienced researcher (TG), the process presented a significant time commitment and may have led to differences in delivery style.

Due to the study timeline and working with school terms and avoiding Ramadan, the intervention was delivered at the height of winter, a contrast to the Australian programme which is delivered only in summer months. This is likely to have impacted both recruitment and intervention attendance.

The session observations, and feedback from participants and facilitators were overwhelmingly positive. Despite acknowledged limitations, the adapted HDHK programme was delivered and received well. The observed outcome measure of weight change across the programme was promising and the data collected showed good levels of completion.

Future research

At this time the Trial Steering Committee did not recommend progressing to a full RCT in the context in which this study was set. However, there may be other settings within the UK in which some of the barriers that were faced would either not occur, or could be addressed or ameliorated. Another avenue which could be explored would be for some of the education sessions to be delivered online which has been shown to be successful in some groups[45] and would reduce the time difficulties experienced in delivering content in the face to face sessions. In line with the recommendations from the study PPI group, delivery of the programme outside the context of weight management may result in higher recruitment.

CONCLUSIONS

The majority of difficulties experienced in this study stem from the setting and context under which it was to be delivered, and whilst we do not recommend progressing this feasibility study

For peer review only - http://bmjopen.bmj.com/site/about/guidelines.xhtml

to a full RCT, we recognise that the outcomes and recommendations made may have been different had the study been trialled in a different setting. A number of challenging circumstances converged within the study which likely compounded further the difficulties encountered in delivery: recruitment difficulties, the untimely change in facilitation staff and the poor weather throughout recruitment and delivery. The intervention was rated highly by those who attended and the weight loss achieved by the intervention participants was promising, although no definitive conclusions can be drawn owing to the small sample size.

Figure 1: Participant Flow through HDHK-UK for fathers

Author contribution

KJ, PA, AB, CC, AD, EF, LJ, MP, MS, MY and PM conceived the study; KJ was PI; YS undertook the statistical analysis, PH was senior statistician; TG was study co-ordinator and led the process evaluation; LJ was the qualitative lead; TG and KH undertook the qualitative interviews and analysis; PM and CC conceived and designed the original HDHK intervention; PM and MY advised on training and delivery; CC and EM advised on dietary assessment; AE was PPI lead; all authors interpreted the findings; TG and KJ drafted the manuscript; all authors and commented on paper drafts and agreed the final version.

Competing interests

Professors Philip Morgan and Clare Collins designed the original Healthy Dads, Healthy Kids Programme in Australia. The other authors declare no competing interests.

Acknowledgements

We would like to thank all participating families, the local authorities for their support and direction and the facilitators who delivered the programme. We acknowledge members of the study steering committee: Professor John Wright, Bradford Institute for Health Research (Chair); Professor Pat Hoddinott, University of Stirling; Elaine Nicholls, Keele University; Mr Ray Fiveash, PPI representative. We acknowledge Kathy Jones and Cassius Campbell who delivered the training programme and Jeremy Davies from the Fatherhood Institute. We thank Jeszemma Garratt from the Fatherhood Institute and the Parent Advisory Panel and staff who contributed to the research delivery: Dr Khaled Ahmed, Felicity Brant, David Sardar and Meanaz Akhtar. We acknowledge academics from the University Newcastle, Australia who developed the original Healthy Dads, Healthy Kids programme and who supported the study through the development of training materials and shared their experience of delivery in the Australian setting – Professor David Lubans and Kristen Saunders.

For peer review only - http://bmjopen.bmj.com/site/about/guidelines.xhtml

Funding

Study funding was granted in October 2015 by the National Institute of Health Research Public Health Research programme (Ref 14/185/13); KJ is part-funded by NIHR Collaborations for Leadership and Health Research and Care West Midlands. The views expressed are those of the authors and not necessarily those of the NHS, the NIHR, or the Department of Health and Social Care.

Trial registration

ISRCTN registry January 2017: (ISRCTN16724454.https://doi.org/10.1186/ISRCTN16724454).

Ethical approval

Ethical approval for the two phases of the study was obtained from The University of Birmingham Science, Technology, Engineering and Mathematics Ethical Review Committee (16th January 2017 (ethics reference; ERN_16-1323).

Data sharing: All data are available on request from the corresponding author

REFERENCES

1. *The NHS Long Term Plan* London: NHS; 2019.

2. Kopelman P. Health risks associated with overweight and obesity. *Obes Rev* 2007;**8 Suppl 1**:13-7. http://dx.doi.org/10.1111/j.1467-789X.2007.00311.x

3. Prospective Studies Collaboration. Body-mass index and cause-specific mortality in 900 000 adults: collaborative analyses of 57 prospective studies. *The Lancet* 2009;**373**:1083-96. http://dx.doi.org/https://doi.org/10.1016/S0140-6736(09)60318-4

4. Public Health England. *Health Matters: Obesity and the food environment.* Public Health England; 2017. URL: https://www.gov.uk/government/publications/health-matters-obesity-and-the-food-environment/health-matters-obesity-and-the-food-environment--2 (Accessed 28/05/2018).

5. National Statistics. *Statistics on Obesity, Physical Activity and Diet. England 2017.* . National Statistics; 2017. URL: https://files.digital.nhs.uk/publicationimport/pub23xxx/pub23742/obes-phys-acti-diet-eng-2017-rep.pdf. (Accessed 28/05/2018).

6. NHS Digital. *Health Survey for England 2016. Adult overweight and obesity.* . NHS Digital; 2016. URL: https://digital.nhs.uk/data-and-information/publications/statistical/health-survey-for-england/health-survey-for-england-2016 (Accessed 28/05/2018).

7. National Institute for Health and Clinical Excellence. *BMI: preventing ill health and premature death in black, Asian and other minority ethnic groups. Public health guideline [PH46]*; 2013.

For peer review only - http://bmjopen.bmj.com/site/about/guidelines.xhtml

8. Gettler LT, Sarma MS, Gengo RG, Oka RC, McKenna JJ. Adiposity, CVD risk factors and testosterone: Variation by partnering status and residence with children in US men. *Evolution, Medicine, and Public Health* 2017;**2017**:67-80. http://dx.doi.org/10.1093/emph/eox005

9. Garfield CF, Duncan G, Gutina A, Rutsohn J, McDade TW, Adam EK*, et al.* Longitudinal Study of Body Mass Index in Young Males and the Transition to Fatherhood. *Am J Mens Health* 2016;**10**:Np158-np67. http://dx.doi.org/10.1177/1557988315596224

10. Morgan PJ, Callister R, Collins CE, Plotnikoff RC, Young MD, Berry N*, et al.* The SHED-IT community trial: a randomized controlled trial of internet- and paper-based weight loss programs tailored for overweight and obese men. *Ann Behav Med* 2013;**45**:139-52. http://dx.doi.org/10.1007/s12160-012-9424-z

11. Morgan PJ, Collins CE, Plotnikoff RC, Cook AT, Berthon B, Mitchell S*, et al.* The impact of a workplace-based weight loss program on work-related outcomes in overweight male shift workers. *J Occup Environ Med* 2012;**54**:122-7. http://dx.doi.org/10.1097/JOM.0b013e31824329ab

12. Hunt K, Wyke S, Gray CM, Anderson AS, Brady A, Bunn C*, et al.* A gender-sensitised weight loss and healthy living programme for overweight and obese men delivered by Scottish Premier League football clubs (FFIT): a pragmatic randomised controlled trial. *Lancet* 2014;**383**:1211-21. http://dx.doi.org/10.1016/s0140-6736(13)62420-4

13. Robertson C, Archibald D, Avenell A, Douglas F, Hoddinott P, van Teijlingen E*, et al.* Systematic reviews of and integrated report on the quantitative, qualitative and economic evidence base for the management of obesity in men. *Health Technol Assess* 2014;**18**:v-vi, xxiii-xxix, 1-424. http://dx.doi.org/10.3310/hta18350

14. Young MD, Morgan PJ, Plotnikoff RC, Callister R, Collins CE. Effectiveness of male-only weight loss and weight loss maintenance interventions: a systematic review with meta-analysis. *Obes Rev* 2012;**13**:393-408. http://dx.doi.org/10.1111/j.1467-789X.2011.00967.x

15. Morgan PJ, Young MD, Lloyd AB, Wang ML, Eather N, Miller A*, et al.* Involvement of Fathers in Pediatric Obesity Treatment and Prevention Trials: A Systematic Review. *Pediatrics* 2017;**139**:e20162635. http://dx.doi.org/10.1542/peds.2016-2635

16. Morgan PJ, Collins CE, Plotnikoff RC, Callister R, Burrows T, Fletcher R*, et al.* The 'Healthy Dads, Healthy Kids' community randomized controlled trial: a community-based healthy lifestyle program for fathers and their children. *Prev Med* 2014;**61**:90-9. http://dx.doi.org/10.1016/j.ypmed.2013.12.019

17. Morgan PJ, Lubans DR, Callister R, Okely AD, Burrows TL, Fletcher R*, et al.* The 'Healthy Dads, Healthy Kids' randomized controlled trial: efficacy of a healthy lifestyle program for overweight fathers and their children. *Int J Obes (Lond)* 2011;**35**:436-47. http://dx.doi.org/10.1038/ijo.2010.151

18. Jolly K, Griffin T, Sidhu M, Adab A, Burgess A, Collins CE*, et al.* A weight management programme for fathers of children aged 4-11: cultural adaptation and the Healthy Dads, Healthy Kids UK feasibility RCT. *Public Health Research* in press.

19. Office for National Statistics. *Local Area Report for areas in England and Wales.* . 2017. URL: https://www.nomisweb.co.uk/reports/localarea (Accessed 09/10/2018).

20. Golan M, Weizman A. Familial approach to the treatment of childhood obesity: conceptual mode. *J Nutr Educ* 2001;**33**:102-7.

21. Golan M, Weizman A, Apter A, Fainaru M. Parents as the exclusive agents of change in the treatment of childhood obesity. *Am J Clin Nutr* 1998;**67**:1130-5. http://dx.doi.org/10.1093/ajcn/67.6.1130

For peer review only

22. Bandura A, & National Inst of Mental Health. *Prentice-Hall series in social learning theory. Social foundations of thought and action: A social cognitive theory.* . Englewood Cliffs, NJ, US: Prentice-Hall, Inc.; 1986.

23. Jolly K GT, Sidhu M, Adab P, Burgess A, Collins C, Daley A, Entwistle A, Frew E, Hardy P, Hurley K, Jones L, McGee E, Pallan M, Sun Y, Young M, Morgan P. . A weight management programme for fathers: cultural adaptation of Healthy Dads, Healthy Kids UK and feasibility RCT. *Public Health Research* in press.

24. Department for Communities and Local Government. *The English Indices of Deprivation 2015*. National Statistics; 2015. URL: https://assets.publishing.service.gov.uk/government/uploads/system/uploads/attachment_data/file/465791/English_Indices_of_Deprivation_2015_-_Statistical_Release.pdf (Accessed 09/10/2018).

25. Craig CL, Marshall AL, Sjostrom M, Bauman AE, Booth ML, Ainsworth BE*, et al.* International physical activity questionnaire: 12-country reliability and validity. *Med Sci Sports Exerc* 2003;**35**:1381-95. http://dx.doi.org/10.1249/01.mss.0000078924.61453.fb

26. Furman W, Giberson R. Identifying the links between parents and their children's sibling relationships. In: Shulman S, editor. *Human development, Vol. 7. Close relationships and socioemotional development*Westport, CT, US: Ablex Publishing.; 1995:95-108.

27. Larios SE, Ayala GX, Arredondo EM, Baquero B, Elder JP. Development and validation of a scale to measure Latino parenting strategies related to children's obesigenic behaviors. The parenting strategies for eating and activity scale (PEAS). *Appetite* 2009;**52**:166-72. http://dx.doi.org/10.1016/j.appet.2008.09.011

28. Lampard AM, Nishi A, Baskin ML, Carson TL, Davison KK. The Activity Support Scale for Multiple Groups (ACTS-MG): Child-reported physical activity parenting in African American and non-Hispanic White families. *Behavioral medicine (Washington, DC)* 2016;**42**:112-9. http://dx.doi.org/10.1080/08964289.2014.979757

29. Pan H, Cole T. LMS growth, a Microsoft Excel add-in to access growth references based on the LMS methods. In. 2.77 ed; 2012.

30. McCarthy HD, Jarrett KV, Crawley HF. The development of waist circumference percentiles in British children aged 5.0-16.9 y. *Eur J Clin Nutr* 2001;**55**:902-7. http://dx.doi.org/10.1038/sj.ejcn.1601240

31. Ihmels MA, Welk GJ, Eisenmann JC, Nusser SM. Development and preliminary validation of a Family Nutrition and Physical Activity (FNPA) screening tool. *The International Journal of Behavioral Nutrition and Physical Activity* 2009;**6**:14-. http://dx.doi.org/10.1186/1479-5868-6-14

32. Goodman R. Psychometric Properties of the Strengths and Difficulties Questionnaire. *Journal of the American Academy of Child & Adolescent Psychiatry* 2001;**40**:1337-45. http://dx.doi.org/10.1097/00004583-200111000-00015

33. Herdman M, Gudex C, Lloyd A, Janssen MF, Kind P, Parkin D*, et al.* Development and preliminary testing of the new five-level version of EQ-5D (EQ-5D-5L). *Quality of Life Research* 2011;**20**:1727-36. http://dx.doi.org/10.1007/s11136-011-9903-x

34. Al-Janabi H, N Flynn T, Coast J. Development of a self-report measure of capability wellbeing for adults: the ICECAP-A. *Quality of Life Research* 2012;**21**:167-76. http://dx.doi.org/10.1007/s11136-011-9927-2

35. Stevens K. Developing a descriptive system for a new preference-based measure of health-related quality of life for children. *Quality of Life Research* 2009;**18**:1105-13. http://dx.doi.org/10.1007/s11136-009-9524-9

For peer review only - http://bmjopen.bmj.com/site/about/guidelines.xhtml

36. Stevens K. Working with children to develop dimensions for a preference-based, generic, pediatric, health-related quality-of-life measure. *Qualitative Health Research* 2010;**20**:340-51.

37. Stevens K. Assessing the performance of a new generic measure of health-related quality of life for children and refining it for use in health state valuation. *Applied Health Economics and Health Policy* 2011;**9**:157-69. http://dx.doi.org/10.2165/11587350-000000000-00000

38. Gale NK, Heath G, Cameron E, Rashid S, Redwood S. Using the framework method for the analysis of qualitative data in multi-disciplinary health research. *BMC Medical Research Methodology* 2013;**13**:117-. http://dx.doi.org/10.1186/1471-2288-13-117

39. Mobley A, Gans K, Adamsons K, Huedo-Medina T. Healthy Fathers, Healthy Kids: Feasibility of a Father-focused Childhood Obesity Prevention Program for Low-income Families with Preschool Aged Children (OR13-03-19). *Current developments in nutrition* 2019;**3**:nzz050.OR13-03-19. http://dx.doi.org/10.1093/cdn/nzz050.OR13-03-19

40. Jolly K, Lewis A, Beach J, Denley J, Adab P, Deeks JJ*, et al.* Comparison of range of commercial or primary care led weight reduction programmes with minimal intervention control for weight loss in obesity: Lighten Up randomised controlled trial. *BMJ* 2011;**343**. http://dx.doi.org/10.1136/bmj.d6500

41. British Heart Foundation. *The National Audit of Cardiac Rehabilitation (NACR) annual statistical report 2017*; 2018.

42. Pallan M, Griffin, T., Hurley, K.L., Lancashire, E., Blissett, J., Frew, E., Griffith, L., Hemming, K., Jolly, K., McGee, E., Thompson, JL., Jackson, L., Gill, P., Parry, J., Adab, P. Cultural adaptation of an existing children's weight management programme: the CHANGE intervention development and feasibility RCT. . *Health Technology Assessment* 2018.

43. Pagoto SL, Schneider KL, Oleski JL, Luciani JM, Bodenlos JS, Whited MC. Male Inclusion in Randomized Controlled Trials of Lifestyle Weight Loss Interventions. *Obesity* 2012;**20**:1234-9. http://dx.doi.org/doi:10.1038/oby.2011.140

44. NICE. *Weight management: lifestyle services for overweight or obese adults: Public Health Guidleine [PH53]*; 2014.

45. Mensorio MS, Cebolla-Marti A, Rodilla E, Palomar G, Lison JF, Botella C*, et al.* Analysis of the efficacy of an internet-based self-administered intervention ("Living Better") to promote healthy habits in a population with obesity and hypertension: An exploratory randomized controlled trial. *Int J Med Inform* 2019;**124**:13-23. http://dx.doi.org/10.1016/j.ijmedinf.2018.12.007

For peer review only - http://bmjopen.bmj.com/site/about/guidelines.xhtml

Figure 1: Participant Flow through HDHK-UK for fathers

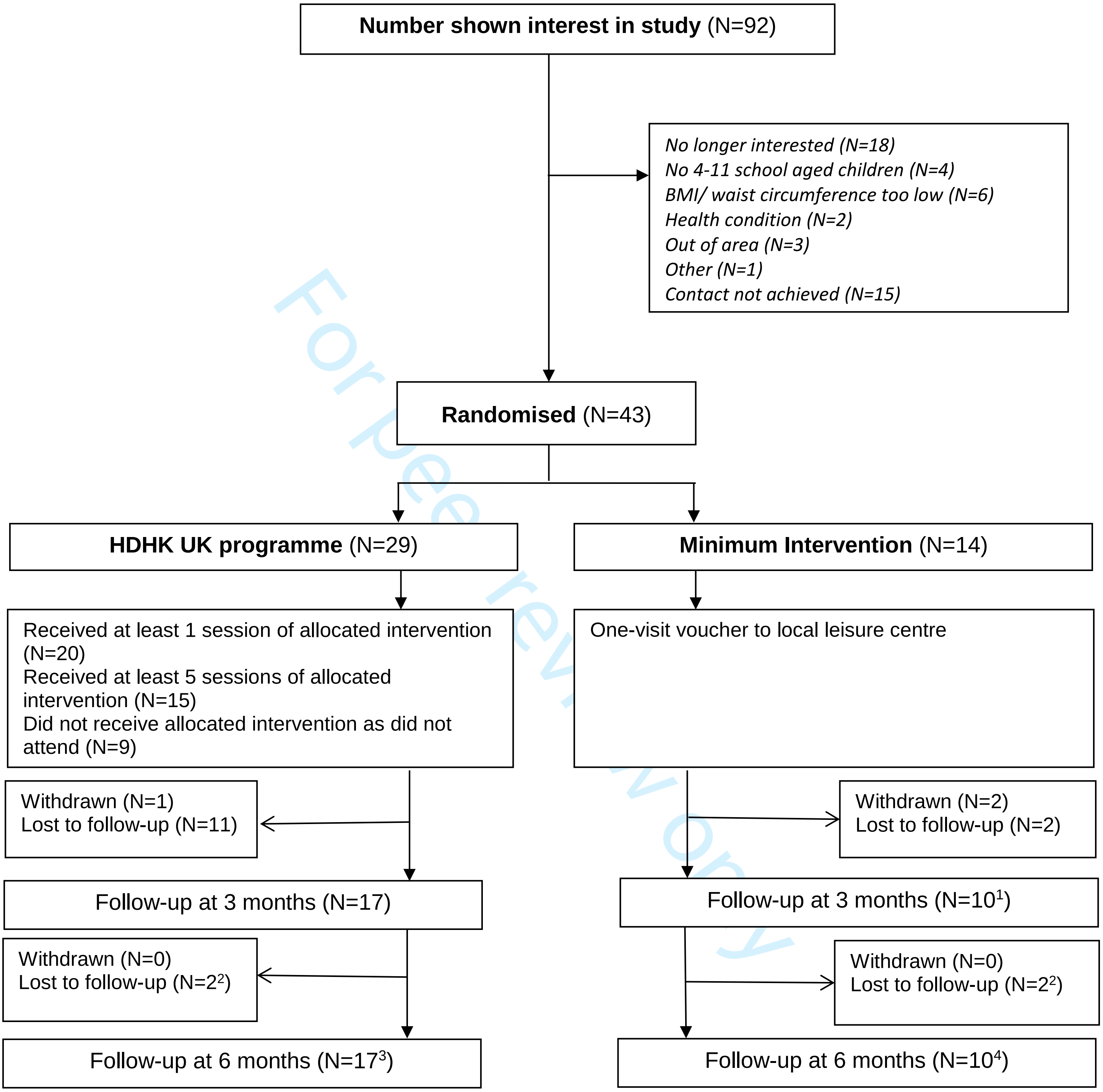

[1] One participant completed questionnaire only;
[2] Lost to follow up out of those participants who completed follow up at month 3;
[3] 15 participants continued from the previous 17 who completed the follow up at month 3; 2 participants returned from lost to follow up at month 3;
[4] 8 participants continued from the 10 who completed follow up at month 3, 2 participants returned from lost to follow up at month 3.

For peer review only - http://bmjopen.bmj.com/site/about/guidelines.xhtml

Web-table 1: The HDHK UK programme reported according to the Template for Intervention Description and Replication (TIDieR) checklist

Item	Item description
1. Name	Healthy Dads, Health Kids UK
2. Why	Following the success of the Healthy Dads, Healthy Kids programme in Australia the aim of adapting the programme was to test the suitability of the programme to an ethnically diverse UK population.<br><br>The primary aim of adapting the HDHK programme was to increase acceptability of the programme to a socio-economically disadvantaged, ethnically diverse UK population.
3 & 4: Materials and Procedures	Fathers of primary school aged children who were classed as overweight or obese (BMI >25kg/m^2 (>23kg/m^2 for fathers of South Asian origin) with primary school aged children (4-11 years) were invited to take part in the HDHK study. Participants randomised to the intervention group were asked to attend the HDHK intervention programme with their children. The intervention programme involved a 90 minute session once a week for nine weeks with weekly goals and activities to be completed in their own time<br><br>**Materials provided to the participants**<br><br>Fathers and children were provided with an intervention pack:<br><br>**Fathers:**<br>- A handbook to accompany the father-only education sessions<br>- A logbook for monitoring of weekly activities including step counter, weight chart etc.<br>- A HDHK branded black T-shirt<br>- Nine 'Green slips' - cards with weekly activities to be completed either on their own, with their children or as a family<br>- A certificate (presented by their children) on completion of the programme<br><br>**Children:**<br>- A workbook to work through as part of the children-only education sessions. This included weekly activities to complete with their father<br>- Activity cards with instructions how to practice fundamental movement skills and a spinner (as part of a game to select which card to pick each day)<br>- A HDHK branded green T-shirt<br>- A certificate (presented by facilitators) on completion of the programme<br><br>**Materials provided to the facilitators**<br><br>- HDHK branded red T-shirt<br>- Facilitators guide to intervention delivery

For peer review only - http://bmjopen.bmj.com/site/about/guidelines.xhtml

	- Father-only education session facilitators were provided with the PowerPoint slides for delivery<br>- Access to on-line demonstrations of the physical activity components of the programme<br>- Exercise<br><br>Each week the 90 minute course followed the same format:<br><br>**0-15 minutes. Father and children review of their weekly goals with facilitators**<br><br>- A review session where participants and their children updated the facilitators on their activity and if they had completed their weekly tasks the children were given a sticker for their workbooks<br>- This section also included a father-child bonding activity where they were encouraged to talk to each other or play a short game such as 'thumb war' or guess each other's favourite movie/food/activity.<br>- Fathers had opportunity to use scales to weigh themselves and complete their log book<br><br>**15-45 minutes. Fathers ('father-only') and children ('children-only') participate in separate education sessions**<br><br>- Father only sessions were delivered by the facilitator using a pre-prepared PowerPoint slide show which was delivered encouraging discussion around the key points. There were also additional activities each week to be completed in the logbook such as setting goals (weight, physical activity of nutrition goals), calculating daily energy needs,<br>- Every week fathers were asked to report on their weight and average number of daily steps (aiming to 10,000 steps/day)<br>- Child-only education sessions were delivered by working through the children's workbook. The session content followed that of the father sessions but made simple to be child appropriate.<br>- Children were also asked to select two activities each week to complete with their fathers. The activities were planned around physical activity or nutrition, such as 'how many press ups can Dad do in 1minute', 'set up an obstacle course', 'get Dad to drink at least 8 glasses of water a day'.<br><br><u>Fathers' sessions:</u><br><br>Week 1: Dads matter in children's health - Highlights the unique influence of dads in contributing to the physical and mental health of children.<br><br>Week 2: Weight management for men - Explores the challenges of healthy eating in the modern world, outlines the mathematics of weight loss and setting SMART goals to achieve activity and dietary ambitions.<br><br>Week 3: Being a healthy dad – Strategies to enhance your and your family's life. Highlights 9 weight loss tips for men, tells dads

For peer review only

	how to 'stay on track' and provides advice on sustainable approaches to weight loss.<br><br>Week 4: Healthy eating for families (Mums attend) - Provides advice on appropriate portion sizes for the whole family, discusses strategies for implementing the trust paradigm to encourage their children to eat healthily at home.<br><br>Week 5: The unique and powerful influence of fathers - Explains to dads why they have such a powerful influence over their kids, the importance of being a good role model and outlines the most effective parenting style.<br><br>Week 6: Raising active kids in an inactive world - Explains the growing issues of childhood obesity and why physical activity is so important for kids, highlights key strategies for dads to be physical activity leaders.<br><br>Week 7: 'Switching on' your child's mind by 'Switching off' - Highlights the physical and mental health issues created by excessive screen time and provides strategies for 'switching off'.<br><br>Week 8: 'Healthy' fathering in a busy world - Encourages discussion of barriers and solutions for achieving SMART goals, highlights opportunities to create family traditions and maximize the time dads can spend with their kids.<br><br>Children's sessions:<br><br>Week 1: Rough and tumble play - children learn about their mission to 'get dad fit and healthy' and are taught about rough and tumble activities.<br><br>Week 2: Turning Dad into a healthy eater - Through fun activities, kids learn about 'sometimes' foods and 'anytime' foods and how they can encourage dad to eat more healthily.<br><br>Week 3: Helping dad make healthy choices - children came up with suggestions for their Dad's packed lunch and were introduced to goal setting.<br><br>Week 4: The HDHK rainbow plate - Through fun activities, children learn about different fruits and vegetables and are challenged to make their plates 'rainbows' with a variety of healthy fruits and vegetables.<br><br>Week 5: Fun times with Dad - children are given activities to help them think about games they can play with dad to spend quality time together.<br><br>Week 6: Let's get active - children learning about importance of being active and considering how to stay active.<br><br>Week 7: Helping Dad 'Switch off' - children think about activities they could enjoy with Dad instead of playing on the computer or watching TV.

For peer review only

For peer review only - http://bmjopen.bmj.com/site/about/guidelines.xhtml

	Week 8: Becoming Dad's personal trainer - children develop an activity board with games and exercises the family can complete at home.<br><br>Week 9: Helping dad stay on track<br>**45-90 minutes. Fathers and children joint physical activity session**<br><br>Each week the joint father and child physical activity session covered three components:<br>- Rough and Tumble Play – *hands on activities played by each participant with their child, such as 'sock wrestle' (trying to get each other's socks off), 'stand up Dad' (children would try to stop their Dad standing up)*<br>- Fundamental skill development (for children) – *Coached activities focusing on child skill development of FMS such as catching, throwing, jumping, running etc.*<br>- Fitness activity (for fathers) – *Fitness activity focusing on fathers' fitness with support from children (e.g. Child helps count Dad's press ups, or sits on Dads back to make it harder or family running games racing each other).*
5. Who provided?	Three facilitators were required to deliver the HDHK programme. One facilitator to deliver the father-only education sessions and two facilitators to deliver to child-only education and joint father and child activity sessions.<br><br>Staff were recruited from four organisations to deliver the programme across the different courses. A face to face training session, a course manual and all materials required for delivery were provided.<br><br>The fathers-only education session facilitators were delivered by:<br>- Local Authority Healthy lifestyle Team (health trainer - female) – course 1<br>- Leisure centre staff employees (health and lifestyles manager and gym manager – both male) – course 2&3<br>- Sports coach of a national coaching organisation (female) – course 4<br><br>For successful delivery father-education facilitators were required to have presentation and group facilitation skills. The programme was designed for this section to be delivered by male facilitators, however due to staff availability this was not always possible.<br><br>The child-only education sessions and joint child and family activity sessions were delivered by:<br>- Self-employed fitness instructor - course 1<br>- Sports coaches – courses 2, 3 & 4.<br><br>Whilst not essential for delivery, sports coaching skills supported successful delivery to the physical activity elements of the programme. For child protection and ease of facilitation it was recommended that two facilitators ran the child-only education sessions. The second facilitator does not need to lead the session

For peer review only

For peer review only - http://bmjopen.bmj.com/site/about/guidelines.xhtml

	and this role could be undertaken by research staff or work experience students.
6: How	HDHK-UK is a group based programme, optimum group size 8-15 fathers
7. Where?	The programme is delivered in community venues such as community, youth or leisure centres and schools. The venue must have classroom (or equivalent) space for an educational session with PowerPoint projection facilities and a sports hall (or equivalent) for the physical activity session.
8. When and how often?	The programme is 9-weeks long with one 90-minute session delivered each week. Sessions need to be delivered at a time fathers and their children (aged 4-11 years) can attend. Of the four courses delivered, two were on a weekday evening, two were on a weekend. The weekend sessions were more popular.
9. Tailoring	Each week throughout the education session fathers were encouraged to reflect on the session content and apply the key messages in context to their own personal circumstances. Similarly, participants were asked to tailor their weekly goals and set home activities to meet their own and their family needs.
Modifications	Details of the programme adaptation from the original Australian programme are presented earlier in this chapter. Throughout the delivery of the adapted programme small modifications were made between courses, mainly where delivery of the father-only education session took longer than the allocated 30 minutes the material was shortened to ensure there was sufficient time for the physical activity session.
How well the programme was delivered -planned	The university research team completed observations of intervention delivery, completing a checklist to report on items of fidelity, including session timings, the delivery of key messages, facilitator skills and enthusiasm and participants responsiveness.
How well the programme was delivered - actual	Researcher observations of programme delivery showed that it was delivered with good fidelity. The main challenge was keeping the father-only education session to the allotted 30-minutes. This was improved throughout the courses. The fidelity of intervention delivery is described in Chapter 6.

For peer review only

Web-table 2: Baseline characteristics for the completers and non-completers at 6 months

	**Non-completers at 6 months** (N=16)	**Completers at 6 months** (N=27)	**Overall** (N=43)
**Minimisation Variables**			
Ethnicity			
White British	10 (62.5%)	7 (25.9%)	17 (39.5%)
Non White British	6 (37.5%)	20 (74.1%)	26 (60.5%)
**Demographics**			
Age (years)			
Mean (SD), N	38.7 (7.7), 16	40.7 (5.5), 27	40.0 (6.4), 43
Minimum-Maximum	23.6-56.0	31.6-52.8	23.6-56.0
Weight (kg)			
Mean (SD), N	93.4 (16.0), 16	89.2 (14.9), 27	90.8 (15.3), 43
Minimum-Maximum	70.9-136.9	64.0-142.5	64.0-142.5
BMI (kg/m²)			
Mean (SD), N	30.9 (6.1), 16	29.7 (4.4), 27	30.2 (5.1), 43
Minimum-Maximum	25.1-50.9	23.8-45.5	23.8-50.9
Index of Multiple Deprivation quintile, n (%)			
1 (least deprived)	1 (6.3%)	0 (0.0%)	1 (2.3%)

For peer review only - http://bmjopen.bmj.com/site/about/guidelines.xhtml

	**Non-completers at 6 months** (N=16)	**Completers at 6 months** (N=27)	**Overall** (N=43)
2	2 (12.5%)	0 (0.0%)	2 (4.7%)
3	2 (12.5%)	4 (14.8%)	6 (14.0%)
4	2 (12.5%)	7 (25.9%)	9 (20.9%)
5 (most deprived)	9 (56.3%)	14 (51.9%)	23 (53.5%)
Missing	0 (0.0%)	2 (7.4%)	2 (4.7%)

Note: All figures presented are N (%) unless otherwise specified.

For peer review only

CONSORT 2010 checklist of information to include when reporting a randomised trial*

Section/Topic	Item No	Checklist item	Reported on page No
**Title and abstract**			
	1a	Identification as a randomised trial in the title	1
	1b	Structured summary of trial design, methods, results, and conclusions (for specific guidance see CONSORT for abstracts)	2
**Introduction**			
Background and objectives	2a	Scientific background and explanation of rationale	4
	2b	Specific objectives or hypotheses	4
**Methods**			
Trial design	3a	Description of trial design (such as parallel, factorial) including allocation ratio	4
	3b	Important changes to methods after trial commencement (such as eligibility criteria), with reasons	n/a
Participants	4a	Eligibility criteria for participants	4-5
	4b	Settings and locations where the data were collected	4
Interventions	5	The interventions for each group with sufficient details to allow replication, including how and when they were actually administered	5 & supplementary web table
Outcomes	6a	Completely defined pre-specified primary and secondary outcome measures, including how and when they were assessed	6 - 7
	6b	Any changes to trial outcomes after the trial commenced, with reasons	n/a
Sample size	7a	How sample size was determined	5
	7b	When applicable, explanation of any interim analyses and stopping guidelines	n/a
Randomisation:			
Sequence generation	8a	Method used to generate the random allocation sequence	5
	8b	Type of randomisation; details of any restriction (such as blocking and block size)	5
Allocation concealment mechanism	9	Mechanism used to implement the random allocation sequence (such as sequentially numbered containers), describing any steps taken to conceal the sequence until interventions were assigned	5
Implementation	10	Who generated the random allocation sequence, who enrolled participants, and who assigned participants to interventions	5

For peer review only - http://bmjopen.bmj.com/site/about/guidelines.xhtml

Blinding	11a	If done, who was blinded after assignment to interventions (for example, participants, care providers, those assessing outcomes) and how	n/a
	11b	If relevant, description of the similarity of interventions	5
Statistical methods	12a	Statistical methods used to compare groups for primary and secondary outcomes	7 – 8
	12b	Methods for additional analyses, such as subgroup analyses and adjusted analyses	7 – 8
**Results**			
Participant flow (a diagram is strongly recommended)	13a	For each group, the numbers of participants who were randomly assigned, received intended treatment, and were analysed for the primary outcome	8, 9, Figure 1
	13b	For each group, losses and exclusions after randomisation, together with reasons	Figure 1
Recruitment	14a	Dates defining the periods of recruitment and follow-up	5
	14b	Why the trial ended or was stopped	n/a
Baseline data	15	A table showing baseline demographic and clinical characteristics for each group	Table 1
Numbers analysed	16	For each group, number of participants (denominator) included in each analysis and whether the analysis was by original assigned groups	Tables 1 – 3
Outcomes and estimation	17a	For each primary and secondary outcome, results for each group, and the estimated effect size and its precision (such as 95% confidence interval)	Tables 1 – 3
	17b	For binary outcomes, presentation of both absolute and relative effect sizes is recommended	Tables 1 – 3
Ancillary analyses	18	Results of any other analyses performed, including subgroup analyses and adjusted analyses, distinguishing pre-specified from exploratory	n/a
Harms	19	All important harms or unintended effects in each group (for specific guidance see CONSORT for harms)	14
**Discussion**			
Limitations	20	Trial limitations, addressing sources of potential bias, imprecision, and, if relevant, multiplicity of analyses	20 - 21
Generalisability	21	Generalisability (external validity, applicability) of the trial findings	20 – 21
Interpretation	22	Interpretation consistent with results, balancing benefits and harms, and considering other relevant evidence	20-21
**Other information**			
Registration	23	Registration number and name of trial registry	23
Protocol	24	Where the full trial protocol can be accessed, if available	Submitted
Funding	25	Sources of funding and other support (such as supply of drugs), role of funders	22

*We strongly recommend reading this statement in conjunction with the CONSORT 2010 Explanation and Elaboration for important clarifications on all the items. If relevant, we also recommend reading CONSORT extensions for cluster randomised trials, non-inferiority and equivalence trials, non-pharmacological treatments, herbal interventions, and pragmatic trials. Additional extensions are forthcoming: for those and for up to date references relevant to this checklist, see www.consort-statement.org.

For peer review only - http://bmjopen.bmj.com/site/about/guidelines.xhtml
